# Glycolysis gatekeeper PDK1 reprograms breast cancer stem cells under hypoxia

**DOI:** 10.1038/onc.2017.368

**Published:** 2017-11-06

**Authors:** F Peng, J-H Wang, W-J Fan, Y-T Meng, M-M Li, T-T Li, B Cui, H-F Wang, Y Zhao, F An, T Guo, X-F Liu, L Zhang, L Lv, D-K Lv, L-Z Xu, J-J Xie, W-X Lin, E W-F Lam, J Xu, Q Liu

**Affiliations:** 1 Institute of Cancer Stem Cell, Dalian Medical University, Dalian, China; 2 State Key Laboratory of Oncology in South China, Cancer Center, Sun Yat-sen University, Guangzhou, China; 3 Department of Oncology, The First Affiliated Hospital of Dalian Medical University, Dalian, China; 4 Department of Thoracic Surgery, The First Affiliated Hospital of Dalian Medical University, Dalian, China; 5 Department of Pathology, The Second Affiliated Hospital of Dalian Medical University, Dalian, China; 6 Internal Medicine Department of Oncology, the Second Affiliated Hospital of Dalian Medical University, Dalian, China; 7 Dalian Maternal and Child Care Service Centre, Dalian, Liaoning, China; 8 Department of Surgery and Cancer, Imperial College London, London, UK

## Abstract

Glycolysis is critical for cancer stem cell reprogramming; however, the underlying regulatory mechanisms remain elusive. Here, we show that pyruvate dehydrogenase kinase 1 (PDK1) is enriched in breast cancer stem cells (BCSCs), whereas depletion of PDK1 remarkably diminishes ALDH^+^ subpopulations, decreases stemness-related transcriptional factor expression, and inhibits sphere-formation ability and tumor growth. Conversely, high levels of PDK1 enhance BCSC properties and are correlated with poor overall survival. In mouse xenograft tumor, PDK1 is accumulated in hypoxic regions and activates glycolysis to promote stem-like traits. Moreover, through screening hypoxia-related long non-coding RNAs (lncRNAs) in PDK1-positive tissue, we find that lncRNA H19 is responsible for glycolysis and BCSC maintenance. Furthermore, *H19* knockdown decreases PDK1 expression in hypoxia, and ablation of *PDK1* counteracts H19-mediated glycolysis and self-renewal ability *in vitro* and *in vivo*. Accordingly, H19 and PDK1 expression exhibits strong correlations in primary breast carcinomas. H19 acting as a competitive endogenous RNA sequesters miRNA let-7 to release Hypoxia-inducible factor 1α, leading to an increase in PDK1 expression. Lastly, aspirin markedly attenuates glycolysis and cancer stem-like characteristics by suppressing both H19 and PDK1. Thus, these novel findings demonstrate that the glycolysis gatekeeper PDK1 has a critical role in BCSC reprogramming and provides a potential therapeutic strategy for breast malignancy.

## Introduction

Accumulating evidence indicates that a small subpopulation of cancer cells with stem cell properties have a strong correlation with enhanced tumorigenesis, metastasis, relapse and resistance to treatment.^1, 2, 3^ These cancer stem cells (CSCs) display self-renewing capacity and multilineage differentiation into various cell populations within the tumor mass.^4^ Reprogramming of energy metabolism is one of the hallmarks of cancer.^5^ Various studies indicate that deranged metabolism, like aerobic glycolysis, is related to tumor growth and chemoresistance.^6^ CSCs are highly plastic in terms of metabolic machinery and rely on either glycolysis or oxidative phosphorylation (OXPHOS).^7, 8, 9^ As a key glucose metabolism process, glycolysis contributes to CSC maintenance in specific microenvironments, such as hypoxia and nutrient starvation.^10, 11^ Previous studies have revealed that CSCs exhibit a more glycolytic phenotype compared with their differentiated offsprings.^12^ In concordance, glycolysis-associated events/processes, such as glucose uptake, glycolytic enzyme expression, lactate production and ATP levels, are significantly elevated in CSCs, which is also linked to a decrease in mitochondrial oxidative metabolism.^13, 14, 15^ Conversely, inhibition of glycolysis reversely suppresses the CSC maintenance. For example, treatment with the glycolysis inhibitor 3-BrOP notably decreases the side population (SP) in breast cancer cells and eliminate tumorigenesis *in vivo*.^16^ Lactate dehydrogenase A gene (*LDHA)* knockout cancer cells derived from *K*-*Ras* driven non-small cell lung cancer mouse model also display impaired ability to form tumorspheres and tumors.^17^ Together, these studies suggest that glycolysis has a vital role in CSC maintenance, but the underlying mechanisms remain enigmatic and require further investigation.

Pyruvate dehydrogenase kinase 1 (PDK1) phosphorylates the pyruvate dehydrogenase (PDH) E1α subunit and inactivates the PDH enzyme complex that converts pyruvate to acetyl-coenzyme A,^18^ thereby inhibiting pyruvate oxidation via the tricarboxylic acid cycle to generate energy.^19^ As an essential glycolytic enzyme, PDK1 is associated with tumor proliferation, metastasis and poor prognosis.^20, 21, 22^ For example, PDK1 inhibitor (DAP) remarkably suppresses AML cell proliferation, autophagy and increases apoptosis, also eradicates tumor growth in mouse model.^23^ LIN28A/B and let-7g axis regulates the Warburg effect to promote tumor proliferation by targeting PDK1 in Hypoxia-inducible factor (HIF-1)-independent manner.^24^

A recent study has shown that PDK1 is a direct target of oncoprotein HIF-1α,^25^ which regulates *PDK* family and *PKM2* to modulate cell fate reprogramming through early glycolytic shift.^26^ Furthermore, a metabolism proteome analysis delineates induced pluripotent stem cells to be distinct from parental MEFs for displaying high levels of PDK1.^27^ Although these recent findings have indicated that PDK1 has critical roles in regulating tumor progression and stem cell reprogramming, little is known about the mechanism by which PDK1 controls CSC maintenance.

Long non-coding RNAs (lncRNAs) are commonly defined as non-protein- coding transcripts longer than 200 nucleotides. Emerging studies demonstrated lncRNAs acting as oncogenes involved in multiple biological processes during cancer progression.^28, 29^ Notably, numerous lncRNAs participate in cancer cell glucose metabolism regulation. For example, lncRNA NRCP functions as an intermediate binding partner between STAT1 and RNA polymerase II, facilitating the transactivation of downstream target genes, involved in cancer glucose metabolism.^30^ Another finding showed lncRNA UCA1 promotes cancer cell glycolysis through the mTOR-STAT3/microRNA143-HK2 signaling axis.^31^ Equally, much attention has been focused on the regulation of cancer cell stemness by lncRNAs. LncRNA-ROR acts as a competitive endogenous RNA to sponge miRNAs and positively regulate the expression of stem cell-related transcription factors, OCT4, NANOG and SOX2, in human embryonic stem cells.^32, 33^ In addition, oncoprotein Twist is transcriptionally regulated by lncRNA-Hh to directly target GAS1 (growth arrest-specific 1), which promotes the activation of Hh signaling, thereby increasing SOX2 and OCT4 expression to maintain CSC properties.^34^ Collectively, these findings indicate that lncRNAs function as key regulators of CSC glycolysis.

In the present study, we explored the role and regulation of PDK1 in BCSCs and found that PDK1 is required for BCSC reprogramming via activating glycolysis under hypoxic conditions. We identified PDK1 as a downstream target of lncRNA H19, and demonstrated that *PDK1* knockdown markedly inhibits H19-mediated glycolysis and CSC maintenance. Mechanistically, we showed that PDK1 is elevated through the H19/let-7/HIF-1α signaling axis. Intriguingly, we also uncovered that aspirin can suppress glycolysis and BCSC maintenance through repressing H19 and PDK1. Taken together, our studies identify a novel role and regulatory mechanism of PDK1 in BCSC reprogramming, which provides a promising strategy for breast cancer therapy.

## Results

### PDK1 is required for breast cancer stem-like traits

**Figure 1:**
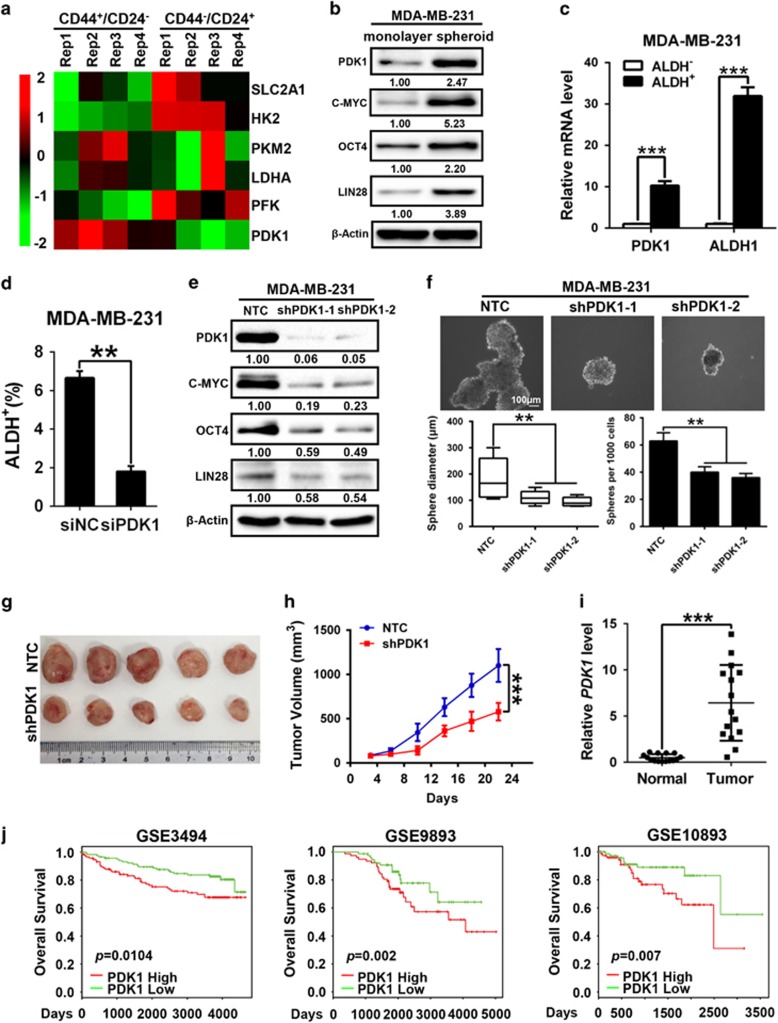
PDK1 is required for breast cancer stem-like properties. (**a**) Heat map displayed the expression signature of candidate genes using genome mRNA expression profiling data (GSE15192) from GEO database, color key indicates log2 values. (**b**) CSC populations in MDA-MB-231 cells were enriched by sphere-formation assay. Expression of candidate proteins (PDK1, C-MYC, OCT4 and LIN28) was analyzed by western blotting. (**c**) Column graph represented the comparison of PDK1 and ALDH1 expression level between ALDH^−^ and ALDH^+^ subpopulations of MDA-MB-231 cells. (**d**) ALDH-positive populations were analyzed following PDK1 interference in MDA-MB-231 cells. (**e**) The protein levels of a panel of stemness-related transcriptional factors were evaluated in *PDK1* knockdown MDA-MB-231 cells. (**f**) Mammosphere formation ability was analyzed following down-regulating of *PDK1* in MDA-MB-231 cells. The representative images were presented (up, scale bar=100 μm) and the diameter and numbers of mammospheres were measured and counted (down). (**g**) Immunodeficient mice (*n*=5) were subcutaneously inoculated with equal number of single cells (1 × 10^6^ cells per mouse). The image was harvested tumors in the end. (**h**) Tumor volumes were monitored as described in materials and methods. (**i**) The expression of *PDK1* in 15 pairs of breast tumors and adjacent normal tissues was subjected to RT-qPCR analysis. (**j**) Kaplan–Meier overall survival plots of breast cancer patients created using PROGgeneV2, data sets from cohort GSE3494, GSE9893, GSE10893. Patients were classified into PDK1-high and PDK1-low subgroups and analyzed as indicated. Data are represented as mean±s.d. **P<0.01, ***P<0.001, *n*=3.

To identify the key glycolytic regulators involved in breast cancer stem-like cell reprogramming, the gene expression patterns of key glycolytic enzymes in the glucose metabolic pathway, including SLC2A1, HK2, PFK, PKM2, lactate dehydrogenase A and PDK1 (Supplementary Figure 1A), were compared between the CD44^+^/CD24^−^ and CD44^−^/CD24^+^ subpopulations from a previously published dataset.^35^ As shown in the heat map of Figure 1a, PDK1 mRNA level in breast cancer stem cell (BCSC) enriched CD44^+^/CD24^−^ subpopulations was substantially higher than that in the control CD44^−^/CD24^+^ fractions, whereas there was no significant increase in the expression of other glycolytic enzymes (Supplementary Figure 1B). To confirm this, we examined PDK1 expression in BCSCs purified from breast carcinoma cells through their mammosphere formation ability and ALDH^+^ cell sorting. Indeed, when compared with other glycolytic enzymes, the mRNA level of PDK1 was evidently higher in spheroids (enriched stemness-related factors MYC, POU5F1 and LIN28) from MDA-MB-231 and MCF-7 cells (Supplementary Figures 1C and D). Moreover, spheroids exhibited much higher protein expression of PDK1, which was associated with higher levels of stemness-related factors in three breast cancer cells (Figure 1b, Supplementary Figures 1E and F). In concordance, ALDH^+^ cells displayed higher PDK1 and ALDH1 mRNA levels compared with ALDH^−^ cells (Figure 1c).

Next, PDK1 was knocked down by siRNA (Supplementary Figure 1G) or shRNA in MDA-MB-231 cells. The stem cell-associated ALDH^+^ population significantly declined following the depletion of PDK1 in MDA-MB-231 cells (Figure 1d). The expression levels of stemness-related factors markedly decreased in PDK1 knockdown cells (Figure 1e). Subsequently, we conducted sphere-formation assay, and observed a significant reduction in spheroid diameters and numbers in PDK1-depleted MDA-MB-231 cells (Figure 1f). Importantly, we performed tumor xenograft assays in nude mice by injecting with control MDA-MB-231-NTC (NTC; non-targeting control) cells or MDA-MB-231-shPDK1 (shPDK1) cells. The results showed that the mice inoculated with shPDK1 cells evidently formed smaller tumor masses than the mice injected with NTC cells (Figures 1g and h), indicating that PDK1 is critical for tumor growth *in vivo*. In addition, PDK1 overexpression cells (PDK1) were established by lentivirus infection in MCF-7 cells. The upregulated PDK1 remarkably increased ALDH^+^ subpopulations, elevated stemness factors expression and promoted sphere-formation ability (Supplementary Figures 1H–J). However, knockdown or overexpression of PDK1 had no effects on cell proliferation, cell cycle progression and cell viability in MDA-MB-231 or MCF-7 cells by BrdU staining (Supplementary Figures 2A and B), cell cycle analysis (Supplementary Figures 2C and D) and CCK8 assay (Supplementary Figures 2E and F), respectively.

Furthermore, we assessed the expression of PDK1 between breast tumor tissues and their adjacent tissues. PDK1 mRNA and protein expression levels were significantly higher in tumor tissues compared with adjacent non-cancerous tissues (Figure 1i and Supplementary Figure 2G). Moreover, the Kaplan–Meier survival analysis demonstrated that high PDK1 levels were a strong indicator for inferior overall survival (Figure 1j) in three patient cohorts (GSE3494, GSE9893 and GSE10893 from GEO data sets), suggesting a significantly unfavorable prognosis and shorter life span. These data provide strong evidence to suggest that PDK1 plays a critical role in the BCSC self-renewal and reprogramming.

### PDK1 activates glycolysis to enhance stemness in hypoxia

**Figure 2:**
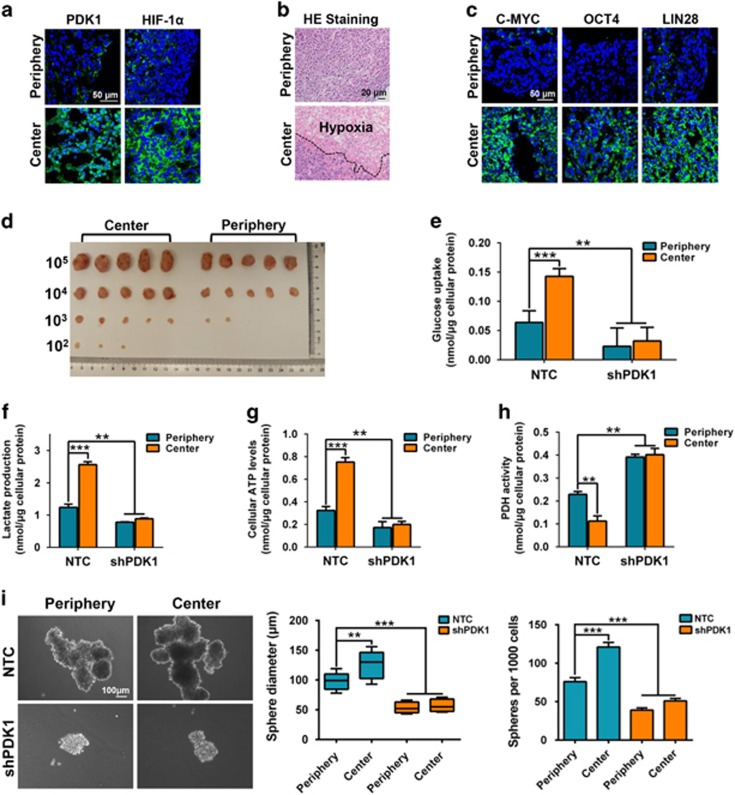
PDK1 activates glycolysis to raise stemness in hypoxia. (**a**) Immunofluorescent staining of PDK1 and HIF-1α in freezed sections (7 mm). DAPI was used as a nuclear staining. The scale bar represents 50 μm. (**b**) Frozen sections of central and periphery regions isolated from xenograft tumors were analyzed by H&E staining. The scale bar represents 20 μm. (**c**) Immunofluorescent staining of stemness-related markers (C-MYC, OCT4 and LIN28) in central and peripheral regions of xenografted tumors. DAPI is used for staining nucleus. The scale bar represents 50 μm. (**d**) Limiting diluted numbers of isolated tumor cells from central and peripheral regions were subcutaneously inoculated into immunodeficient mice (*n*=5). Serial transplantation frequency was analyzed after four weeks. (**e**–**h**) Primary cells isolated from central and peripheral regions of NTC and shPDK1 xenografted tumors were isolated. Intracellular glucose uptake (**e**), lactate production (**f**), cellular ATP levels (**g**) and PDH activity (**h**) were measured and calculated. (**i**) Mammosphere formation ability was analyzed by using isolated cells. The scale bar represents 100 μm. Data shown are mean±s.d. (*n*=3), **P<0.01 and ***P<0.001, respectively.

To further explore whether PDK1 acts as a crucial regulator of BCSC maintenance by modulating glycolysis, we evaluated PDK1-positive cancer cell characteristics using a mouse xenograft tumor model. In this assay, MDA-MB-231 cells were injected subcutaneously in nude mice, and frozen sections of the tumor xenografts were immunofluorescence stained for PDK1 expression. The results showed that PDK1 mostly accumulated in the central regions with highly hypoxic cells as revealed by Hypoxia-inducible factor 1α (HIF-1α) (Figure 2a and Supplementary Figure 3A) and HE staining (Figure 2b), when compared with the peripheral regions. In addition, stemness-related factors, C-MYC, OCT4 and LIN28, were highly expressed in the tumor central regions compared with the peripheral regions (Figure 2c and Supplementary Figure 3B). Next, we digested tissues from tumor peripheric regions (Periphery) and central regions (Center), and the isolated cells were put into primary culture (Supplementary Figure 3C). Central region cells displayed higher PDK1 mRNA level than peripheral cells (Supplementary Figure 3D). Then, tumor xenograft and sphere-formation assays were performed using these primary culture cells. Central region cells again showed higher tumorigenesis rates (Figure 2d) and greater sphere-formation ability (Supplementary Figure 3E) compared with peripheral cells. Moreover, when glycolysis levels in these cells were measured, central cells exhibited higher glucose uptake, lactate production and ATP levels than peripheral cells (Supplementary Figures 3F-H), indicating central cells are addicted to utilizing glycolysis for glucose metabolism. As a direct target of PDK1, PDH activity was lower in central cells (Supplementary Figure 3I).

Next, NTC and shPDK1 MDA-MB-231 cells were injected subcutaneously in nude mice, and the tumors isolated from peripheral and central regions were digested to produce primary culture cells. Consistently, the central cells from the NTC tumors displayed higher levels of PDK1 expression compared with the peripheral cells; however, there were no significant differences in PDK1 levels between peripheral and central cells isolated from shPDK1 tumors (Supplementary Figure 3J). Intriguingly, there were also no significant variations in glycolysis levels, as revealed by their glucose uptake, lactate production, ATP levels, PDH activity (Figures 2e and h) and sphere-formation ability (Figure 2i) between central cells and peripheral cells in shPDK1 tumor compared with NTC group. Together, these data evidently demonstrate that PDK1 activates glycolysis and maintains BCSC properties under hypoxic conditions.

### Hypoxia-induced H19 contributes to glycolysis and stemness in breast cancer

**Figure 3:**
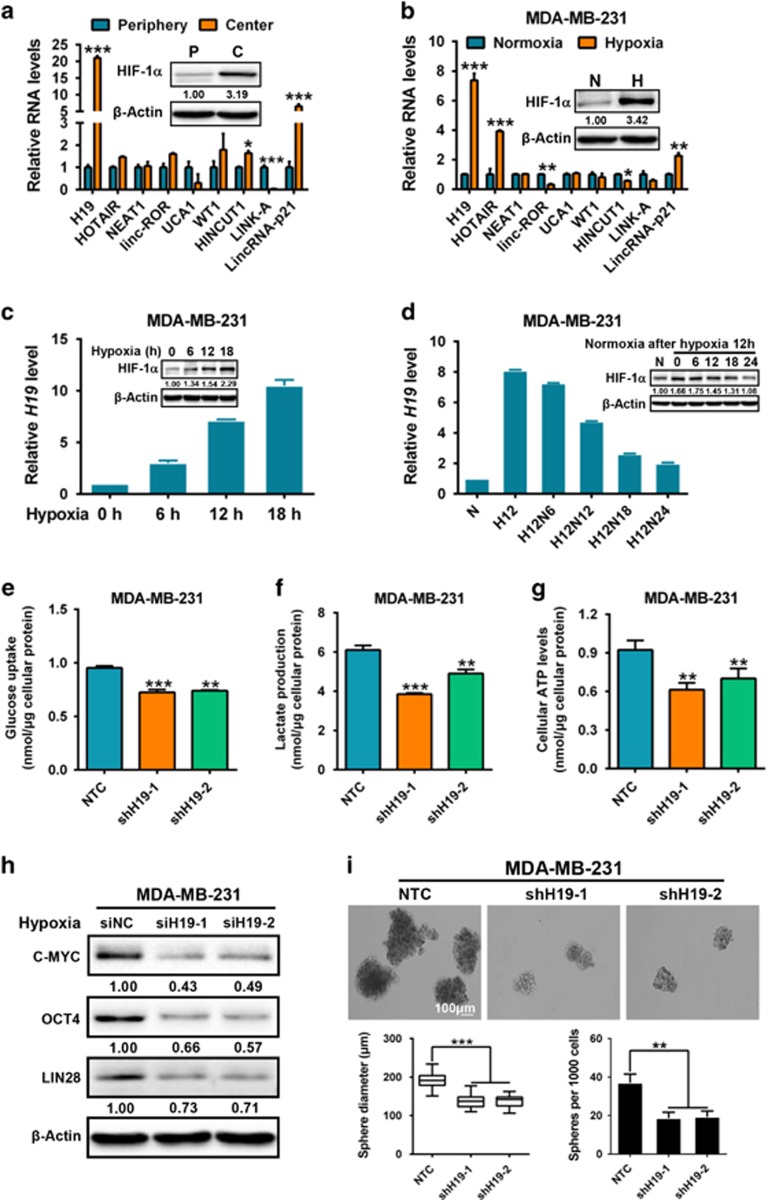
Hypoxia-induced H19 promotes glycolysis and stemness in breast cancer. (**a**) Using cells isolated from peripheral and central regions of xenograft tumors, expression of lncRNAs (H19, HOTAIR, NEAT1, linc-ROR, UCA1, WT1, HINCUT1, LINK-A, LincRNA-p21) was analyzed by RT-qPCR. HIF-1α expression was also analyzed by western blotting. (**b**) MDA-MB-231 cells were cultured under hypoxia condition for 12 h. Expression of lncRNAs (H19, HOTAIR, NEAT1, lnc-ROR, UCA1, WT1, HINCUT1, LINK-A, LincRNA-p21) was analyzed by RT-qPCR. HIF-1α expression was also analyzed by western blotting. (**c**) H19 expression under different hypoxic time points (0, 6, 12 and 18 h) in MDA-MB-231 cells was analyzed by RT-qPCR. HIF-1α protein level was detected by western blotting. (**d**) Analysis of H19 expression under hypoxic condition (12 h) and recovery to normoxia after hypoxia (0, 6, 12, 18 and 24 h) in MDA-MB-231 cells by using RT-qPCR. HIF-1α protein level was detected by western blotting. (**e**–**g**) MDA-MB-231 cells expressing either NTC or shH19 were cultured under hypoxic condition for 24 h. Intracellular glucose uptake (**e**), lactate production (**f**) and cellular ATP levels (**g**) were then measured and normalized based on protein concentration. (**h**) MDA-MB-231 cells transfected with either control siRNA (siNC) or siH19 were cultured under hypoxic conditions. The protein expression of C-MYC, OCT4 and LIN28 was detected after 12 h by western blotting. (**i**) Mammosphere formation ability was analyzed in MDA-MB-231 cells expressing NTC or shH19. Statistics of spheres formation were analyzed after 10 days. The scale bar represents 100 μm. Data were shown as mean±s.d. from triple independent experiments, *P<0.05, **P<0.01 and ***P<0.001, respectively.

As lncRNA can be induced by hypoxia^36^ and is involved in glucose homeostasis^37^ and BCSC maintenance,^38^ these studies prompted us to investigate if lncRNA has a critical role in regulating glycolysis and BCSCs under hypoxia. Using the tumor xenograft primary culture cells. We compared the expression levels of nine hypoxia-related lncRNAs^39, 40, 41^ (H19, HOTAIR, NEAT1, linc-ROR, UCA1, WT1, HINCUT1, LINK-A, LincRNA-p21) between tumor central and peripheral cells. The results showed that H19 is the highest expressing lncRNA in central cells as well as the highest differentially expressed lncRNA when compared with peripheral cells (Figure 3a). Moreover, when breast cancer cells were cultured under hypoxic conditions (O_2_=1%) to mimic the xenograft tumor central microenvironments, H19 expression levels were the highest amongst all lncRNAs studied in both MDA-MB-231 cells (Figure 3b) and MCF-7 cells (Supplementary Figure 4A) under hypoxia. In concordance, the mRNA level of H19 was also the highest expressing lncRNA in BCSC-enriched spheroid cells (Supplementary Figure 4B and C). Indeed, hypoxia elevated H19 expression in a time-dependent manner (Figure 3c and Supplementary Figure 4D), whereas normoxic culture reversed the hypoxia-induced H19 induction in both MDA-MB-231 (Figure 3d) and MCF-7 (Supplementary Figure 4E) cells. Interestingly, *H19* knockdown by two shRNAs (Supplementary Figure 4F and G) caused a significant decrease in cellular glucose uptake, lactate production and ATP levels under hypoxia in both MDA-MB-231 (Figures 3e and g) and MCF-7 cells (Supplementary Figures 4H-J); whereas *H19* knockdown displayed no effects on glycolysis when MDA-MB-231 cells (Supplementary Figures 5A-C) and MCF-7 cells (Supplementary Figures 5D-F) under normoxic conditions. Moreover, silencing of *H19* by siRNA (Supplementary Figures 5G and H) remarkably decreased expression of stemness-related factors under hypoxia in MDA-MB-231 (Figure 3h). In addition, there was a significant reduction in sphere sizes and numbers in MDA-MB-231-shH19 cells (Figure 3i) and MCF-7-shH19 cells (Supplementary Figure 5I). However, knockdown of H19 displayed no significant changes in cell proliferation, cell cycle progression in MDA-MB-231 or MCF-7 cells using BrdU staining (Supplementary Figure 5J) and cell cycle analysis (Supplementary Figure 5K). These results demonstrate that hypoxia-induced H19 participates in glycolysis and CSC maintenance in breast cancer.

### Depletion of PDK1 antagonizes H19-mediated glycolysis and stemness

**Figure 4:**
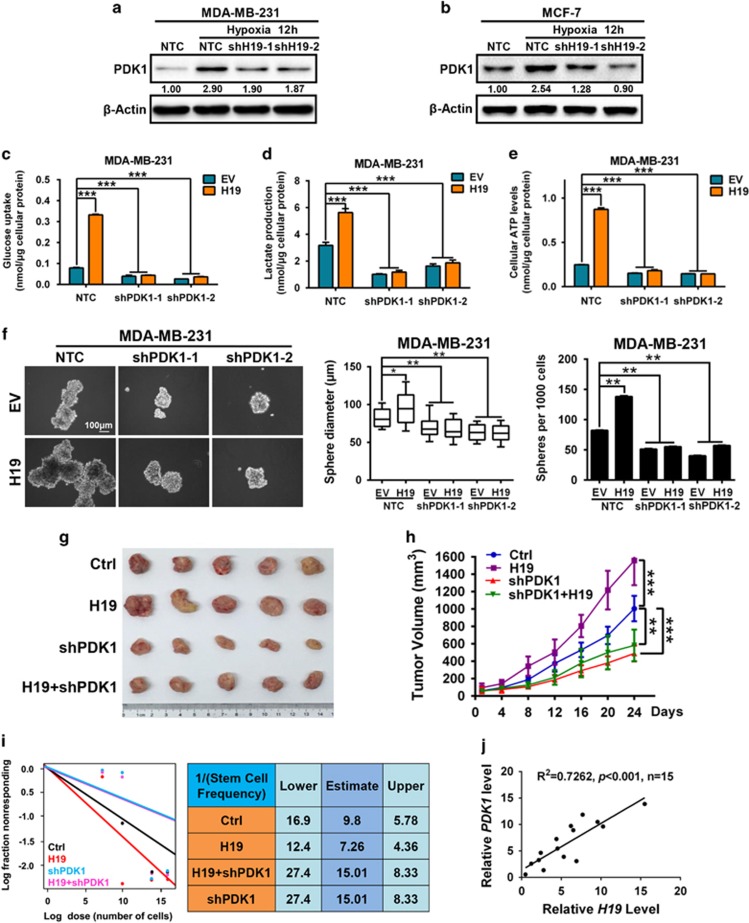
Depletion of PDK1 counteracts H19-mediated glycolysis and stemness. (**a**–**b**) MDA-MB-231 and MCF-7 cells expressing either NTC or shH19 were cultured under hypoxia for 12 h compared with NTC cells cultured in normoxia. PDK1 expression was detected by western blotting. (**c**–**e**) MDA-MB-231 cells expressing either NTC or shPDK1 were infected with lentivirus expressing H19 and empty vector (EV) for establishing stable cells. Intracellular glucose uptake (**c**), lactate production (**d**) and cellular ATP levels (**e**) were then measured and normalized based on protein concentration. (**f**) Mammosphere formation ability was analyzed, the scale bar represents 100 μm. (**g** and **h**) Immunodeficient mice (*n*=5) were subcutaneously inoculated with equal number of single cells (5 × 10^5^ cells per mice) (**g**) and tumor volume were monitored after 24 days (**h**). (**i**) *In vivo* limiting dilution assays performed by plating decreasing numbers of primary xenografted tumor cells (H19, shPDK1 and H19 plus shPDK1) into immunodeficient mice (*n*=5) calculated with extreme limiting dilution assay analysis (left); right panel: stem cell frequencies were estimated as the ratio 1/*x* with the upper and lower 95% confidence intervals, where 1=stem cell and *x*=all cells. (**j**) There was a significant correlation of PDK1 and H19 mRNA levels in breast patient samples (*n*=15, *R*^2^=0.7262, *P*<0.001; linear regression analysis). Data shown are mean±s.d. (*n*=3), *P<0.05, **P<0.01 and ***P<0.001, respectively.

To identify further whether PDK1 is the potential downstream targets of H19, we first examined PDK1 expression upon H19 knockdown in breast cancer cells. Results showed that ablation of *H19* significantly decreased the expression of PDK1 induced by hypoxia in MDA-MB-231 (Figure 4a) and MCF-7 cells (Figure 4b). Similarly, the mRNA levels of PDK1 were downregulated in MDA-MB-231-shH19 cells and MCF-7-shH19 cells under hypoxia (Supplementary Figure 6A). Loss of *H19* also resulted in a substantial increase of PDH activity in both MDA-MB-231 and MCF-7 cells (Supplementary Figure 6B) under hypoxic condition. In order to verify whether H19-mediated glycolysis levels and BCSC maintenance are dependent on PDK1, H19-stably overexpressing (H19) cells were established from shPDK1 or NTC MDA-MB-231 cells and MCF-7 cells (Supplementary Figures 6C and D). H19 overexpression significantly promoted glucose uptake, lactate production and ATP levels, whereas silencing PDK1 reversed this H19-enhanced glycolysis in MDA-MB-231 cells (Figures 4c and e). Similar results were found in MCF-7 cells (Supplementary Figures 6E-G). In addition, PDH activity declined with H19 overexpression, but recovered upon PDK1 deletion in both MDA-MB-231 cell and MCF-7 cells (Supplementary Figure 6H and I). Moreover, H19 overexpression resulted in an increase of tumor sphere-formation capacity in MDA-MB-231 cells, whereas depletion of PDK1 limited this induction (Figure 4f). Similar results were again obtained in MCF-7 cells (Supplementary Figure 6J). Importantly, as continuous tumor growth could be sustained by BCSCs, we performed the serial transplantation assay in nude mice. In the first tumor transplantation, empty vector with NTC (Ctrl), H19 overexpression with NTC (H19), empty vector plus PDK1 knockdown (shPDK1), and H19 plus shPDK1 MDA-MB-231 cells were subcutaneously transplanted into nude mice (*n*=5). The mice injected with H19 cells formed apparently larger tumor masses than the mice injected with Ctrl cells, but shPDK1 markedly reversed the tumor volumes (Figures 4g and h), indicating PDK1 was critical for H19-mediated tumor growth. In addition, H19 significantly promoted the secondary limited dilution tumor transplantation, whereas PDK1 depletion substantially reversed tumorigenic ability (Figure 4i). Furthermore, H19 and PDK1 levels also displayed a significant correlation in patient tumor samples (Figure 4j, *n*=15). These results strongly support the notion that PDK1 is one of key downstream targets of H19-mediated glycolysis and CSC maintenance.

### H19 enhances PDK1 expression in a HIF-1α-dependent manner

**Figure 5:**
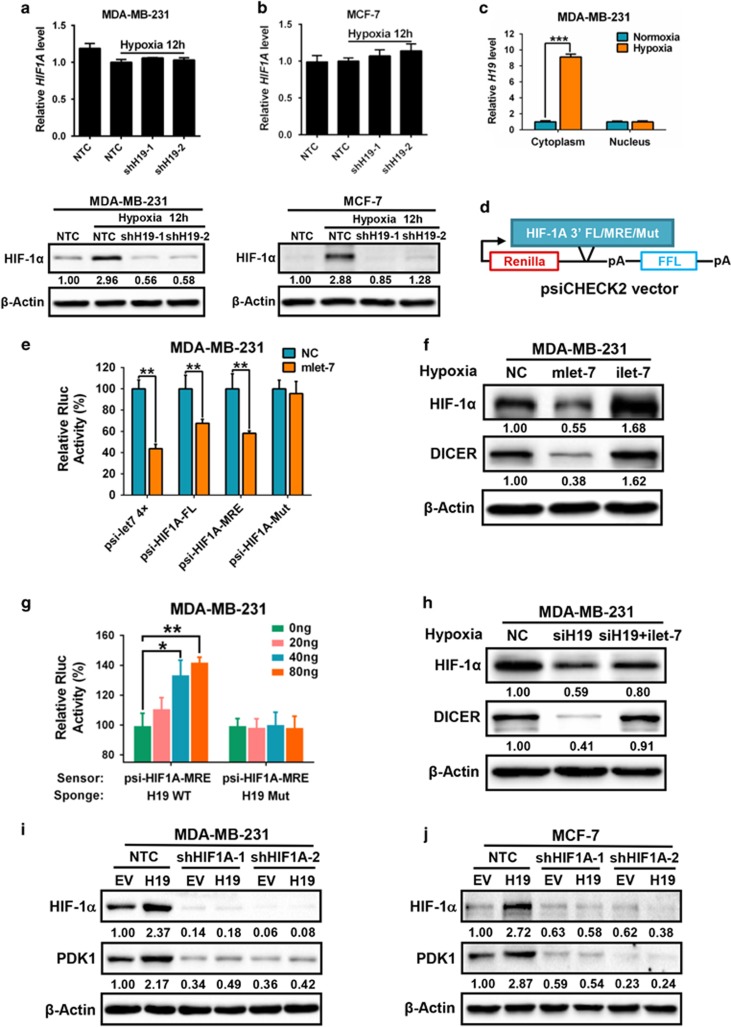
H19 elevates PDK1 expression in a HIF-1α-dependent manner. (**a**, **b**) MDA-MB-231 and MCF-7 cells expressing either NTC or shH19 were cultured under hypoxic condition for 12 h. HIF-1α mRNA and protein expression levels were detected by RT-qPCR (up) and western blotting (down). (**c**) MDA-MB-231 cells were cultured under normoxic or hypoxic conditions for 12 h. The mRNA level of H19 in cytoplasm and nucleus were detected by RT-qPCR. (**d**) Diagram represents the let-7 putative binding site on 3′UTR of *HIF1A*, this site was inserted to the cloning site of psiCHECK2 vector. (**e**) The psi-HIF1A-FL, MRE, Mut and psi-let-7 4x, HIF-1α and let-7a, let-7b vectors were co-transfected into MDA-MB-231 cells and the regulation of HIF-1α by let-7a and let-7b was studied by luciferase assay. (**f**) HIF-1α expression was measured in MDA-MB-231 cells with let-7 mimics (mlet-7) and let-7 inhibitors (ilet-7). DICER is a target of let-7 and used as a positive control. (**g**) MDA-MB-231 cells were transfected with let-7 sensor (psi-HIF1A-MRE) together with 0, 20, 40 and 80 ng of wild-type H19 (WT) or mutant H19 (Mut) plasmids. Dual-luciferase reporter activity was analyzed. (**h**) HIF-1α expression was detected in siH19 and siH19 plus let-7 inhibitor compared with negative control in MDA-MB-231 cells. DICER is a target of let-7 and used as a positive control. (**i**, **j**) MDA-MB-231 and MCF-7 cells expressing either NTC or shHIF1A were infected with vector expressing H19 and control vector. PDK1 and HIF-1α expression was detected by western blot. Data shown are mean±s.d. (*n*=3), *P<0.05, **P<0.01 and ***P<0.001, respectively.

We next investigated the molecular mechanism whereby H19 regulates PDK1 expression. As PDK1 is a validated target of HIF-1α, whereas H19 and HIF-1α are both induced in hypoxia, it is plausible that H19 regulates PDK1 via HIF-1α in hypoxia. To test this conjecture, the expression of HIF-1α was firstly examined in *H19* knockdown cells. The result showed that although there were no significant changes in mRNA levels of HIF-1α in both MDA-MB-231 and MCF-7 cells upon *H19* silencing (Figure 5a and b, up), HIF-1α protein expression was markedly decreased in shH19 cells under hypoxia (Figures 5a and b, down). In addition, H19 was highly induced in the cytoplasm but not in the nucleus under hypoxia (Figure 5c and Supplementary Figure 7A). These findings suggested that H19 might interact with miRNAs in the cytoplasm and functions as an endogenous sponge for miRNAs, which will in turn lead to increased HIF-1α expression. As H19 is a sponge of let-7,^42^ the *HIF1A* 3′UTR full-length sequence (FL) including the putative miRNA (let-7) response element (MRE) were cloned into the psiCHECK2 vector, and the MRE was mutated in psiCHECK2-Mut vector (Figure 5d and Supplementary Figure 7B), respectively. Then, psiCHECK2-HIF1A-FL, MRE, Mut and psiCHECK2-let-7 4 × (harboring four let-7-binding sites; used as a positive control) were transfected into MDA-MB-231 and MCF-7 cells together with mlet-7 (let-7 mimics) in parallel with negative controls. The results showed that mlet-7 significantly repressed the relative luciferase activity of reporter psi-HIF1A-FL and MRE, whereas had no effects on psi-HIF1A-Mut (Figure 5e and Supplementary Figure 7C). In addition, as DICER is a known target of let-7,^43^ both DICER and HIF-1α protein levels (Figure 5f and Supplementary Figure 7D) were decreased in the presence of mlet-7 and increased in the presence of let-7 inhibitors, but the mRNA levels had no significant changes (Supplementary Figure 7E). Moreover, when we co-transfected psi-HIF1A-MRE (sensor) with increasing amounts of wide-type H19 (WT, sponge of let-7) into MDA-MB-231 and MCF-7 cells, the relative luciferase activity was promoted in response to WT H19, but not by H19 with mutated let-7 binding site (Mut H19) in a dose-dependent manner (Figure 5g and Supplementary Figure 7F). Consistently, let-7 released by siH19 decreased DICER and HIF-1α expression, which could be rescued by let-7 inhibitors in MDA-MB-231 cells (Figure 5h) and MCF-7 cells (Supplementary Figure 7G). Furthermore, stable HIF1A knockdown cells were established (Supplementary Figure 7H) and displayed a decrease in mRNA levels as expected under hypoxia (Supplementary Figure 7I). Importantly, knockdown HIF1A could reverse H19-elevated PDK1 expression in both MDA-MB-231 (Figure 5i) and MCF-7 cells (Figure 5j). These results reveal that PDK1 is regulated through the H19/let-7/HIF-1α axis.

### Aspirin suppresses glycolysis and stemness maintenance by inhibiting H19 and PDK1

**Figure 6:**
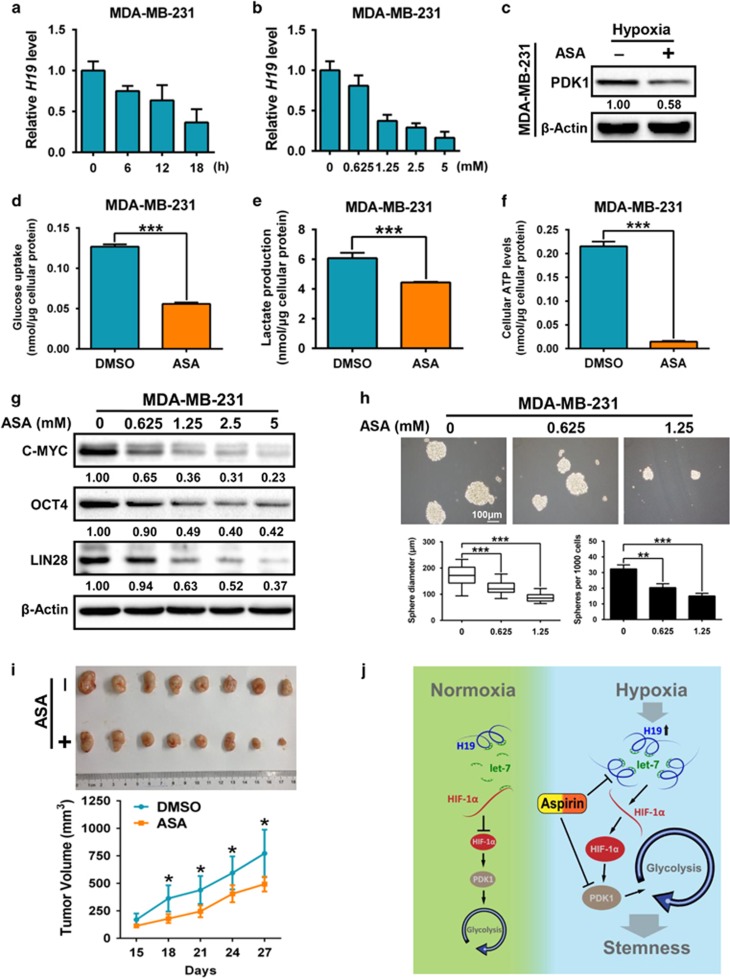
Aspirin suppresses glycolysis and stemness maintenance through dampening H19 and PDK1. (**a**) MDA-MB-231 cells were treated with aspirin (5mm) under hypoxic condition. Expression of H19 was detected by RT-qPCR at different time points (0, 6, 12 and 18 h). (**b**) MDA-MB-231 cells were treated with aspirin under hypoxic condition for 12 h. Expression of H19 was detected by RT-qPCR in aspirin increasing doses (0, 0.625, 1.25, 2.5 and 5 mm). (**c**) MDA-MB-231 cells were treated with aspirin under hypoxic condition for 48 h. PDK1 expression was detected by western blotting. (**d**–**f**) MDA-MB-231 cells were treated with aspirin under hypoxic condition for 24 h. Intracellular glucose uptake (**d**), lactate production (**e**) and cellular ATP levels (**f**) were measured. (**g**) MDA-MB-231 cells were treated with aspirin at different doses (0, 0.625, 1.25, 2.5 and 5 mm) for 12 h. Expression of BCSC markers (C-MYC, OCT4, LIN28) was analyzed by western blotting. (**h**) MDA-MB-231 cells were treated with aspirin for 12 days and mammosphere forming ability was analyzed. The scale bar represents 100 μm. (**i**) Immunodeficient mice (*n*=8) were subcutaneously inoculated with equal number of MDA-MB-231 (1 × 10^6^ cells per mouse). Aspirin was taken orally at 10 days after transplanting. Tumor xenografts were monitored for four weeks. The representative image (up) and growth curve (down) were shown. (**j**) Model depicts PDK1 reprograms breast cancer stem-like cells under the hypoxia. Data shown are mean±s.d. (*n*=3), * *P*<0.05, ** *P*<0.01 and *** *P*<0.001, respectively.

As aspirin (acetylsalicylic acid) could effectively block generation of BSCs and inhibit glycolysis,^44, 45^ we next treated MDA-MB-231 and MCF-7 cells with aspirin to determine whether it can regulate glycolysis by targeting H19 and PDK1. The results showed that aspirin markedly decrease H19 levels in both time- (Figure 6a and Supplementary Figure 8A) and dose- (Figure 6b and Supplementary Figure 8B) dependent manner. Under hypoxia, the expression of PDK1 was also eliminated by aspirin in MDA-MB-231 (Figure 6c) and MCF-7 cells (Supplementary Figure 8C). Conversely, the PDH activity was remarkably increased by treatment with aspirin (Supplementary Figures 8D and E). Interestingly, aspirin significantly inhibited glycolysis by decreasing cellular glucose uptake, lactate production and ATP levels in both MDA-MB-231 (Figures 6d and f) and MCF-7 cells (Supplementary Figures 8F-H). Furthermore, aspirin also caused a dose- and time-dependent reduction in the expression of stemness-related factors, including C-MYC, OCT4 and LIN28 (Figure 6g and Supplementary Figure 8I) in MDA-MB-231 cells as well as mammosphere numbers and diameters in MDA-MB-231 (Figure 6h) and MCF-7 cells (Supplementary Figure 8J). Importantly, xenograft mice subcutaneously injected with MDA-MB-231 cells and fed with aspirin showed that aspirin restrained tumor growth *in vivo* (Figure 6i). Our data collectively led us to conclude that the glycolysis gatekeeper PDK1 regulated by the H19/let-7/HIF-1α pathway is required for BCSC self-renewal reprogramming in hypoxia, which could be blocked by aspirin (Figure 6j).

## Discussion

In this study, we demonstrate that the glycolytic enzyme PDK1 is required for BCSC reprogramming in hypoxia. Consistently, PDK1 is enriched in BCSC populations and is essential for the maintenance of cancer stem-like properties *in vitro* and *in vivo* (Figure 1). Using mouse xenograft models, PDK1 is found to be highly expressed in tumor hypoxic regions and promotes glycolysis to maintain stemness (Figure 2). Moreover, the lncRNA H19 is to facilitate glycolysis and breast cancer stemness under hypoxia (Figure 3). Here, we describe a mechanism in which the hypoxia-induced H19 functions as a competitive endogenous RNA to sponge miRNAs, such as let-7, thereby relieving the expression of HIF-1α and, ultimately leading to the increase in PDK1 expression (Figures 4 and 5). Importantly, aspirin can effectively restrict BCSCs by decreasing both H19 and PDK1 expression (Figure 6).

Reprogramming is referred to as the conversion of differentiated cells to a stem-like state. Ectopic expression of four transcription factors (Oct4, Klf4, Sox2 and c-Myc) reprograms various types of somatic cells to induced pluripotent stem cells.^46^ CSC subpopulations have high expression of self-renewal genes (for example, MYC and SOX2).^47^ In our study, knockdown or overexpression of PDK1 markedly decreased or increased the expression of stemness-related transcriptional factors (c-MYC, OCT4 and LIN28) respectively, indicating PDK1 acts as a key factor in BCSC reprogramming.

The metabolic phenotype of CSCs has been widely investigated in recent years,^48^ and CSCs have been shown to be primarily glycolytic or preferentially shifted from OXPHOS to glycolysis in a tumor type-dependent manner. For example, tumor-initiating cells isolated from MMTV-Wnt-1 mammary tumors preferentially utilize glycolysis over OXPHOS for energy production, in contrast to non-tumorigenic cancer cells.^12^ In addition, glycolysis is the preferred metabolic process in radio-resistant sphere-forming cells in nasopharyngeal carcinoma^49^ and CD133^+^CD49f^+^ tumor-initiating cells in hepatocellular carcinoma.^50^ Metabolic switch from OXPHOS to glycolysis is required for the characteristics of CD44^+^CD24^low^EPCAM^+^ breast CSCs, due to decreased ROS levels. Consistent with this, the increased glycolysis caused by the loss of FBP1 has been shown to increase breast CSC-like properties and tumorigenesis through promoting the interaction of β-catenin with TCF.^7^ Interestingly, CSCs in some other cancer types utilized OXPHOS as the preferred energy production process, such as the SP cells in lung cancer,^51^ the sphere-forming and CD133^+^ cells for both glioblastoma^52^ and pancreatic ductal adenocarcinoma,^53^ and ROS^low^-quiescent leukemic stem cells.^54^ Although the mechanisms determining the observed OXPHOS phenotype have not yet been fully understood, regulatory proteins of mitochondrial biogenesis and structure could have a crucial role in maintaining stemness properties and functionality.^52, 53^ In our study, we demonstrated that the glycolytic enzyme PDK1 is highly expressed in BCSC populations, including sphere-formation cells and ALDH^+^ cells (Figure 1b and c). In agreement, our results showed that depletion of PDK1 decreased breast cancer cell glycolysis phenotype and thereby depressed BCSC maintenance *in vivo* and *in vitro* (Figure 2). In contrast, recent studies also suggested that PDK1 is associated with tumor proliferation and apoptosis antagonization in glioma cells.^55^ Nevertheless, our results also demonstrated there were no significant changes in breast cancer cell proliferation and cell viability in the two PDK1 knockdown cells (Supplementary Figure 2A-F). Therefore, these findings indicate that BCSC reprogramming regulated by PDK1 is primarily related to self-renewal but not proliferation.

Over the last few years, CSC model has shifted more toward using freshly isolated tumor specimens and early-passage xenografts for transplantation studies rather than cultured tumor cells. There is increased awareness that the xenotransplantation assay is critical for evaluating the existence of cancer stem-like cells.^56^ Hence, the microenvironment and property of PDK1-positive cells were investigated using xenograft mouse models in our study. The immunofluorescence staining on xenograft tumors showed that PDK1 accumulated predominantly in tumor central regions (Figure 2a and b), indicating that the function of PDK1 is related to hypoxia. Moreover, HIF-1α, a vital hypoxia-induced transcriptional factor,^57^ has been shown to promote *PDK1* transcription by binding to consensus core HIF-1α response element in the *PDK1* gene.^25^ In addition, recent studies revealed that numerous hypoxia-induced lncRNAs regulate HIF-1α to contribute to tumor progression. For example, the hypoxia-induced lncRNA-p21 disrupts the von Hippel-Lindau/HIF-1α interaction and declines von Hippel-Lindau-mediated HIF-1α ubiquitination, thereby enhancing HIF-1α-dependent glycolysis and tumorigenesis in cancer cells.^40^ Conversely, lncRNA CPS1-IT1 as a tumor suppressor inhibits cell proliferation, migration and invasion abilities through dampening HSP90, which binds to and activates HIF-1α in hepatocellular carcinoma.^58^ Under normoxic conditions, lncRNA *LINK-A* regulates HIF-1α phosphorylation at Tyr565 and Ser797 by BRK and LRRK2, which promotes HIF-1α stability and its transcriptional activity to induce glycolysis and tumorigenesis.^41^ Our studies showed that the lncRNA H19 is highly expressed under hypoxia through screening hypoxia-related lncRNAs (Figures 3a and b, Supplementary Figure 4A). In addition, the fold change of H19 lncRNA in MDA-MD-231 under hypoxic conditions is significantly higher than that MDA-MB-231 derived from the central region of a xenograft, suggesting that the expression of H19 is regulated by a serial of microenvironmental factors, including hypoxia. Notably, HIF-1α mRNA level did not change after let-7 binding. However, luciferase assays showed let-7 binds to imperfectly complementary sequences in the mRNA resulting in predominant translational repression. Consistent with this, mutation to let-7-binding site disrupts the translational repression conducted by let-7 overexpression (Figure 5e and Supplementary Figure 7C). We are the first to demonstrate that the let-7 miRNA targets 3′-UTR of HIF1A mRNA to decrease HIF-1α protein expression. Thus, the hypoxia-induced H19 functions as a competitive endogenous RNA to sponge let-7, leading to the upregulation of HIF-1α, which promotes PDK1 transcription.

The PDK1 inhibitor dichloroacetate (DCA) has been shown to be effective in many cancer types, including colon and breast cancers and oral squamous cell carcinoma.^59, 60, 61^ In cancer cells, DCA switches the glucose metabolism from aerobic glycolysis to glucose oxidation. This increases the OXPHOS and ROS (reactive oxygen species) production in mitochondria, which limits proliferation and increases apoptosis of cancer cells.^62^ Moreover, DCA also restrains putative CSCs (CD133^+^ and nestin^+^) by increasing mitochondrial ROS production to induce apoptosis in glioblastoma cells.^63^ However, the clinical application of DCA has been limited because of its high toxicities, poor pharmacokinetics, low potency and inferior selectivity.^64^ In particular, a recent study has reported that treatment with DCA even promotes neuroblastoma tumor progression in xenograft mouse model.^65^ As a result, new medications are urgently needed to replace DCA for targeting CSCs. Accumulative evidence has indicated that the anti-inflammatory drug aspirin can exert inhibitory effects on CSCs. For example, aspirin restricts cancer stem-like properties by suppressing self-renewal potential and the expression of stemness-related factors (OCT4, SOX2 and NANOG) in pancreatic cancer and has no significantly toxic effects in normal cells.^66^ In addition, a previous study has showed that aspirin upregulated FOXD3 transcriptionally activate the lncRNA OLA1P2 to block phosphorylated STAT3 homodimer formation to suppress lung tumor metastasis.^67^ Interestingly, our findings were the first to demonstrate that aspirin can significantly decrease lncRNA H19 and PDK1 expression and restrict tumor glycolysis and stemness *in vitro* and *in vivo* (Figure 6 and Supplementary Figure S8). However, the detailed mechanism by which aspirin limits H19 and PDK1 expression needs to be further explored in future studies.

In summary, our findings demonstrate that PDK1 functions downstream of the H19-let-7-HIF-1α-signaling cascade as a metabolic switch to regulate glycolysis, which in turn contributes to BCSC maintenance under hypoxic conditions. In addition, our study also shows that this novel PDK1-mediated CSC regulatory mechanism could be inhibited by the common medicine aspirin, which can provide potential therapeutic opportunities for aggressive breast cancers.

## Materials and methods

### Clinical samples, cell lines and primary breast cancer cell isolation

All breast cancer samples were obtained from newly diagnosed patients with prior patients consent and the approval of the Institutional Clinical Ethics Review Board of the first Affiliated Hospital of Dalian Medical University. Detailed information of samples was presented in Supplementary Table 1. Samples were frozen in liquid nitrogen for mRNA and protein extraction. Human breast cancer cell lines MDA-MB-231, MCF-7, SK-BR-3 and HEK293T were obtained from the American Type Culture Collection (ATCC, Manassas, VA, USA). All cell lines were authenticated by short tandem repeats profiling and tested for mycoplasma contamination. MDA-MB-231, SK-BR-3 and HEK293T cells were cultured in DMEM (Dulbecco’s modified Eagle’s medium, Invitrogen, Carlsbad, CA, USA) supplemented with 10% fetal bovine serum (Gibco, Carlsbad, CA, USA). MCF-7 cells were cultured in DMEM (Invitrogen), supplemented with 0.01 mg/ml of human recombinant insulin (Sigma, St Louis, MO, USA) and 10% fetal bovine serum (Gibco). All cells were maintained in a humidified atmosphere with 5% CO_2_ at 37 °C and were not cultured continuously for >3 months. The cell lines were authenticated at American Type Culture Collection before purchase by their standard short tandem repeat DNA-typing methodology. For primary breast cancer cell isolation, the tumor xenografts were digested with collagenase-hyaluronidase (#07912, Stem Cell Technologies, Shanghai, China) for 2 h at 37 °C. Following this, the cells were treated for 3 mins with trypsin-ethylenediaminetetraacetic acid, washed and seeded in 10% FBS DMEM. After culture in a 5% CO_2_ incubator at 37 °C for 12 h, the cells were treated for 1 min with trypsin-ethylenediaminetetraacetic acid. Then stroma cells were washed away by phosphate-buffered saline. Tumor cells still attached in the petri dish and culture for another 24 h. Tumor cells without stroma were harvested and prepared for subsequent experiments.

### Measurement of lactate production, glucose uptake, ATP production and PDH activity

The extracellular lactate was measured using the cell culture medium with Lactate Colorimetric Assay Kit (BioVision, Milpitas, CA, USA) according to the manufacturer’s instruction. Intracellular glucose was measured using cell lysates with Glucose Colorimetric/Fluorometric Assay Kit (BioVision) according to the manufacturer’s instruction. ATP levels were measured using an ATP Colorimetric/Fluorometric Assay Kit (Sigma) according to the manufacturer’s instructions. PDH activity was measured using PDH Activity Colorimetric Assay Kit (BioVision) according to the manufacturer’s instructions. The values were normalized to the protein concentration.

### Animal studies

All animal studies were approved by the Institute Animal Care and Use Committee of Dalian Medical University, and carried out in accordance with established institutional guidelines and approved protocols. For xenograft transplantation assay, female BALB/c nude mice (4–6 weeks old) were purchased from Beijing Vital River Laboratory Animal Technology Co. Ltd. To evaluate tumor growth in mouse models, 100 μl of cell suspension (1 × 10^6^ cells) in phosphate-buffered saline containing 50% Matrigel (BD Biosciences, CA, USA) were subcutaneously inoculated into the back of the mouse. Tumor formation was monitored for 22 days. The tumor sizes were measured periodically and calculated using the formula=0.5 × *a* × *b*^2^ (*a* and *b* were the long and short diameter of the tumors, respectively). For limiting dilution assay, isolated single-cell suspensions were prepared from tumor xenografts. Equal amounts of cells were serially diluted from 1 × 10^5^ to 1 × 10^2^ cells per 100 μl in phosphate-buffered saline containing 50% Matrigel (BD Biosciences, Bedford, MA, USA), and then subcutaneously injected into nude mice (4–6 weeks old). Tumor formation was monitored for four weeks. The tumor sizes were measured periodically and calculated using the formula=0.5 × a × *b*^2^ (*a* and *b* were the long and short diameter of the tumors, respectively). Limiting dilution analysis was calculated using the Extreme Limiting Dilution Analysis software (http://bioinf.wehi.edu.au/software/elda/).^68^

For the animal studies presented in Figure 6i, MDA-MB-231 cells (5 × 10^5^) were subcutaneously injected into BALB/c nude male mice (4–6 weeks old, *n*=8). On day 10, the mice were randomly distributed into two groups and intragastrically administered aspirin or vehicle control once daily. After administering the drug or vehicle for 18 days, the mice were killed and the tumor xenografts were immediately dissected, the tumor volumes were measured by calipers once every three days, estimated using the formula=0.5 × *a* × *b*^2^ (*a* and b were the long and short diameter of the tumors, respectively).

### Statistical analysis

Cohort data sets were downloaded from NCBI. R language (R3.1.0) and PROGgeneV2 were used for calculation of gene expression and survival analysis.^69^ Statistical analysis of the results was performed by using the GraphPad Prism software (La Jolla, CA, USA). The data were expressed as mean±s.d. Kaplan–Meier statistics and log-rank (one tail) test were performed to estimate the significance of differences in overall survival of patients among different groups. All other *P*-values were obtained using Student's *t*-test.

For RNA extraction and quantitative RT-PCR assays, fluorescence-activated cell sorting, mammosphere formation assay, western blot analysis, Plasmids construction and stable cell lines generation, siRNAs microRNA mimics and microRNA inhibitors transfection, BrdU staining assay, cell cycle analysis, cell viability assay, immunofluorescent staining, hematoxylin and eosin staining of tissue sections, luciferase reporter assay, see Supplementary Information.
