# Erratum: Glycolysis gatekeeper PDK1 reprograms breast cancer stem cells under hypoxia

**DOI:** 10.1038/onc.2017.407

**Published:** 2017-12-18

**Authors:** F Peng, J-H Wang, W-J Fan, Y-T Meng, M-M Li, T-T Li, B Cui, H-F Wang, Y Zhao, F An, T Guo, X-F Liu, L Zhang, L Lv, D-K Lv, L-Z Xu, J-J Xie, W-X Lin, E W-F Lam, J Xu, Q Liu

**Keywords:** Long non-coding RNAs, Breast cancer, Cancer metabolism, Cancer stem cells

**Correction to:** *Oncogene* (2018) **37**, 1062–1074; doi: 10.1038/onc.2017.368; published online 6 November 2017

This article was originally published under NPG's License to Publish, but has now been made available under a CC BY license. The PDF and HTML versions of the paper have been modified accordingly.

