# Supplementary material for: Glycolysis gatekeeper PDK1 reprograms breast cancer stem cells under hypoxia
Source: Oncogene. 2017 Nov 6;37(8):1062–74. doi: 10.1038/onc.2017.368 (PMC5851116; doi:10.1038/onc.2017.368)
Supplement: Supplementary Material [file onc2017368x1.docx]

**Supplementary Figure Legends**

*Supplementary Figure 1 (related to Figure 1)*

(A) Schematic illustration of key glucose metabolic steps and relevant enzymes. (B) Column graph represented the comparison of *SLC2A1*, *HK2*, *PKM2*, *LDHA*, *PFK and PDK1* expression values between CD44^+^/CD24^−^ and CD44^−^/CD24^+^ subpopulations in the dataset (GSE15192). (C) CSC populations of MDA-MB-231 cells were enriched using sphere formation assay. Expression of candidate genes (*SLC2A1*, *HK2*, *PKM2*, *LDHA*, *PFK, PDK1, MYC, POU5F1 and LIN28*) was analyzed by RT-qPCR. (D and E) CSC populations were enriched by sphere formation assay in MCF-7 cells. (D) Expression of candidate genes (*SLC2A1*, *HK2*, *PKM2*, *LDHA*, *PFK, PDK1, MYC, POU5F1 and LIN28*) was analyzed by RT-qPCR. (E) Expression of proteins (PDK1, C-MYC, OCT4, LIN28) was analyzed by western blot. (F) SK-BR-3 cells CSC-enriched populations were enriched by sphere formation assay. Expression of proteins (PDK1, C-MYC, OCT4, LIN28) was analyzed by Western blot. (G) Knockdown efficiency of PDK1 was measured after transfection with siRNA targeting PDK1 in MDA-MB-231 cells. (H-J) PDK1 overexpressed cells were established in MCF-7 cells. (H) ALDH-positive populations were analyzed; (I) Expression of proteins (PDK1, C-MYC, OCT4, LIN28) was analyzed by Western blotting; (J) Mammosphere formation ability was analyzed following overexpression PDK1. The representative images were presented (scale bar=100μm) and the diameter and numbers of mammospheres were measured and counted. Data shown are mean±SD (n=3), * *p* < 0.05, ** *p* < 0.01 and *** *p* < 0.001, respectively.

*Supplementary Figure 2 (related to Figure 1)*

(A-B) Cell proliferation was determined by measuring BrdU incorporation using flow cytometry. (C-D) Cell cycle was analyzed by propidium iodide staining and measured by flow cytometry. Sub-G0/G1 phase, S phase and G2/M phase were analyzed using flow cytometry. (E-F) Growth rates of breast cancer cells were measured by CCK-8 viability assay. (G) PDK1 expression of three pairs of breast tumors (T) and adjacent normal tissues (N) was subjected to western blot analysis. Data are represented as mean±SD. Comparison was analyzed by two-tailed Student’s t-tests, n.s. represents no significance, n=3.

*Supplementary Figure 3 (related to Figure 2)*

MDA-MB-231 cells were subcutaneously injected into nude mice. (A) PDK1 and HIF-1α positive cells and total populations in peripheral and center regions of xenografted tumor were counted (B) C-MYC, OCT4 and LIN28 positive cells and total populations in peripheral and center regions of xenografted tumor were counted (C) Schematic diagram showing tumor isolated from tumor peripheral regions and central regions to form primary cultures. (D) PDK1 mRNA level was detected by RT-qPCR in peripheral and central cells. (E) Mammosphere formation was analyzed with peripheral and central cells. The representative images (left, scale bar=100μm), sphere diameter (middle) and sphere numbers (right) were shown. (F-I) Intracellular glucose uptake (F), lactate production (G), ATP levels (H) and PDH activity (I) were measured in cells isolated from central and peripheral regions of xenograft tumor. (J) Expression of PDK1 in peripheral and central cells from NTC and shPDK1 tumors was analyzed by RT-qPCR. Data are represented as mean±SD. **p* < 0.05, ***p* < 0.01, *** *p* < 0.001, n=3.

*Supplementary Figure 4 (related to Figure 3)*

(A) MCF-7 cells were cultured under hypoxic conditions for 12 hours. Expression of lncRNAs (H19, HOTAIR, NEAT1, linc-ROR, UCA1, WT1, HINCUT1, LINK-A and LincRNA-p21) was analyzed by RT-qPCR. (B and C) CSC populations were enriched by sphere formation assay in MDA-MB-231 and MCF-7 cells. Expression of candidate lncRNAs (H19, HOTAIR, NEAT1, linc-ROR, UCA1, WT1, HINCUT1, LINK-A and LincRNA-p21) was analyzed by RT-qPCR in spheroid and monolayer cells. (D) H19 expression under different hypoxic time points (0, 6, 12 and 18 hours) in MCF-7 cells was analyzed by RT-qPCR. HIF-1α protein levels were detected by western blot. (E) Analysis of H19 expression under hypoxic condition (12 hours) and recovery to normoxia after hypoxia (0, 6, 12, 18 and 24 hours) in MCF-7 cells by using RT-qPCR. HIF-1α protein level was detected by western blot. (F and G) Knockdown efficiency of H19 was measured after infection with lentivirus harboring shRNA targeting H19 in MDA-MB-231 and MCF-7 cells. (H-J) Intracellular glucose uptake (H), lactate production (I) and cellular ATP levels (J) were measured in NTC and shH19 MCF-7 cells under hypoxic conditions for 24 hours. Data are represented as mean±SD. n.s. means no significance, **p* < 0.05, ***p* < 0.01, *** *p* < 0.001, n=3.

*Supplementary Figure 5 (related to Figure 3)*

(A-C) Intracellular glucose uptake (A), lactate production (B) and cellular ATP levels (C) were measured in NTC and shH19 MDA-MB-231 cells under normal conditions for 24 hours. (D-F) Intracellular glucose uptake (D), lactate production (E) and cellular ATP levels (F) were measured using NTC and shH19 MCF-7 cells that cultured under normal conditions for 24 hours. (G and H) Knockdown efficiency of H19 was measured after transfection with two siRNAs targeting H19 in MDA-MB-231 and MCF-7 cells. (I) Mammosphere formation ability was analyzed in MCF-7 cells expressing NTC and shH19. Statistics of sphere formation were analyzed after 10 days. The scale bar represents 100μm. (J) Cell proliferation was determined by measuring BrdU incorporation using flow cytometry in MDA-MB-231 and MCF-7 cells. (K) Cell cycle was analyzed by propidium iodide staining and measured by flow cytometry. Sub G0/G1 phase, S phase and G2/M phase were analyzed using flow cytometry in control and H19-knockdown MDA-MB-231 and MCF-7 cells. Data are represented as mean±SD. n.s. means no significance, **p* < 0.05, ***p* < 0.01, *** *p* < 0.001, n=3.

*Supplementary Figure 6 (related to Figure 4)*

(A-B) MDA-MB-231 cells and MCF-7 cells expressing either NTC or shH19 were cultured under hypoxic conditions for 12 hours. PDK1 mRNA level (A) and PDH activity (B) were measured. (C-D) MDA-MB-231 and MCF-7 cells expressing either NTC or shRNA were infected with vector expressing H19 and empty vector for establishing stable cells. PDK1 expression was detected by Western blotting. (E-G) Using MCF-7 cells expressing EV plus NTC, shPDK1, H19 and H19 plus shPDK1. Intracellular glucose uptake (E), lactate production (F), cellular ATP level (G) were measured. (H-I) Using MDA-MB-231 and MCF-7 cells expressing NTC plus EV, shPDK1, H19 and H19 plus shPDK1, PDH activity was measured. (J) Using MCF-7 stable cells, mammosphere formation ability was analyzed, the scale bar represents 100 μm. Data are represented as mean±SD. **p* < 0.05, ***p* < 0.01, *** *p* < 0.001, n=3.

*Supplementary Figure 7 (related to Figure 5)*

(A) MCF-7 cells were cultured under normoxic or hypoxic conditions for 12 hours. The mRNA levels of H19 in cytoplasmic and nuclear were detected by RT-qPCR. (B) Putative let-7 MRE sequence predicted online was shown (upper panel); MRE wide-type (MRE) sequence and mutated sequence (Mut) were shown (lower). (C) Using psiCHECK2-HIF1A-FL, MRE, Mut vectors and let-7 mimic, the regulation of HIF-1α by let-7a and let-7b studied by luciferase assay in MCF-7 cells. (D) HIF-1α expression was measured in MCF-7 cells with let-7 mimics (mlet-7) and let-7 inhibitors (ilet-7). DICER is a target of let-7 and used as a positive control. (E) MDA-MB-231 and MCF-7 cells were transfected with either mlet-7 or ilet-7. HIF1A mRNA was analyzed by RT-qPCR. (F) let-7 sensor (psiCHECK2-HIF1A-MRE) was transfected into MCF-7 cells, together with 0, 20, 40 and 80 ng of sponge plasmid wide-type H19 (WT) or mutant H19 (Mut). (G) HIF-1α expression was detected in siH19 and siH19 plus let-7 inhibitor compared to negative control in MCF-7 cells. DICER is a target of let-7 and used as a positive control. (H-I) MDA-MB-231 and MCF-7 cells expressing NTC or shHIF1A, HIF1A and PDK1 mRNA levels were analyzed by RT-qPCR. Data are represented as mean±SD. **p* < 0.05, ***p* < 0.01, *** *p* < 0.001, n=3.

*Supplementary Figure 8 (related to Figure 6)*

(A) MCF-7 cells were treated with aspirin (5mM) under hypoxia. Expression of H19 was detected by RT-qPCR in different time points (0, 6, 12 and 18 hours). (B) MCF-7 cells were treated with aspirin under hypoxia for 12 hours. Expression of H19 was detected by RT-qPCR with increasing doses (0, 0.625, 1.25, 2.5 and 5mM) of aspirin. (C) MCF-7 cells were treated with aspirin under hypoxic condition for 48 hours. PDK1 expression was detected by western blot. (D and E) PDH activity was measured in MDA-MB-231 and MCF-7 cells treated with aspirin (5mM) under hypoxia for 24 hours. (F-H) MCF-7 cells were treated with aspirin (5mM) under hypoxic condition for 24 hours. Intracellular glucose uptake (F), lactate production (G), cellular ATP level (H) were measured. (I) MDA-MB-231 cells were treated with aspirin (5mM) for different days (0, 1, 3 and 5 days). Expression of stemness-related factors (C-MYC, OCT4 and LIN28) was analyzed by Western blotting. (J) Mammosphere formation ability was analyzed in MCF-7 cells treated with aspirin (5mM) for 12 days (Scale bar=100μm). Data are represented as mean±SD. *** *p* < 0.001, n=3.

**Supplementary Materials and Methods**

*RNA extraction and Real Time quantitative -PCR (RT-qPCR) assays*

Total RNA was extracted by TRIzol reagent (Invitrogen, Carlsbad, CA, USA) according to the manufacturer’s protocol. The cDNA was generated with an oligo-dT primer by EasyScript One-Step gDNA Removal and cDNA Synthesis SuperMix Kit (TransGene Biotech, Beijing, China). Real-time quantitative PCR was performed using the specific SYBR Select Master Mix (Invitrogen, Carlsbad, CA, USA) in an MX3000p cycler (Stratagene, La Jolla, CA, USA). Changes of mRNA levels were determined by the 2^-△△^CT method using Actin for internal crossing normalization. Detailed primer sequences for RT-PCR and qPCR were listed in Supplementary Table 1.

*Fluorescence activated cell sorting*

For ALDH1 assay, the ALDH^+^ population was detected by ALDEFLUOR kit (Shanghai Stem Cell Technology Co. Ltd, Shanghai, China) following manufacturer instructions. MDA-MB-231 cells (1×10^6^/mL) were analyzed on a BD FACS calibur flow cytometer (Ann Arbor, MI, USA) after staining in ALDH1 substrate containing assay buffer for 30 minutes at 37°C. The negative control was treated with diethylaminobenzaldehyde (DEAB), a specifc ALDH1 inhibitor. ALDH^+^ or ALDH^-^ cells (at least 1×10^6^) were collected for RNA extraction.

*Mammosphere formation assay*

For mammosphere formation assay, single cells were seeded at a density of 1×10^3^ cells/ml in ultralow attachment 6-well plates with serum-free DMEM/F12 (Gibco, Carlsbad, CA, USA) supplemented with 2% (v/v) B27 (Invitrogen, Carlsbad, CA, USA), 20ng/ml EGF (Sigma, St Louis, MO, USA), 20ng/ml basic fibroblast growth factor (bFGF, BD Biosciences, CA, USA), and 4μg/ml Heparin (Sigma, St Louis, MO, USA) for 10 to 14 days. The mammospheres were counted and photographed using inverted fluorescence microscope (Ix81, Olympus, Japan). The diameters of the mammospheres were calculated with the CellSens Dimension software (Ix81, Olympus, Japan).

*Western blot analysis*

The western blot procedure was performed as described previously[^1^](#_ENREF_1) with some modifications. Briefly, cells were harvested and lysed in a lysis buffer containing a cocktail of protease inhibitors. After centrifugation at 12000 rpm for 15 minutes at 4°C, supernatants were collected and used for western blot. Equal amounts of protein extract were electrophoresed in 10% SDS-PAGE gels and then transferred to nitrocellulose membranes (Millipore, USA). The membranes were blocked with 5% bovine serum albumin (BSA, Sigma, St Louis, MO, USA) at room temperature (RT) for 1 hour, incubated with the primary antibody overnight at 4°C. After incubation with peroxidase-conjugated secondary antibodies (Thermo Scientific, Rockford, lL, USA) for one hour at room temperature. The membranes were washed in Tris-buffered saline with 0.1% Tween-20 and proteins were visualized by chemiluminescence (Amersham, Marlborough, MA, USA). Western blots were visualized using a Bio-Rad XRS autoimager (BioRad, Hercules, California, USA). Relative intensities of bands were quantified by software Image Lab 4.0.1 (BioRad, Hercules, California, USA) normalized to β-Actin. The information of antibodies was listed as follows: β-Actin (Proteintech, Wuhan, China), C-MYC (Cell Signaling Technology, Danvers, MA, USA), OCT4 (Cell Signaling Technology, Danvers, MA, USA), LIN28 (Abcam, Cambridge, MA, USA), PDK1 (Cell Signaling Technology, Danvers, MA, USA), HIF-1α (Cell Signaling Technology, Danvers, MA, USA).

*Plasmids construction and stable cell lines generation*

Wild-type human H19 (WT H19) and mutant human H19 (Mut H19) were constructed as previously described.^2^ psiCHECK2-let7 4× was built as described by Iwasaki et al.^3^ psiCHECK2-FL: HIF1A-3’UTR-full length was obtained by PCR then inserted into the l Xho1 and Not1 sites of the luciferase reporter vector psiCHECK2 (Promega, Madison, WI, USA). The primer sequences were CGCTCGAGGCTTTTTCTTAATTTCATTCC (Forward) and TAGCGGCCGCGCCT

GGTCCACAGAAGATGTT (Reverse). psiCHECK2-MRE: the unique putative let-7 miRNA response element (MRE) of *HIF1A* was predicted by bioinformatics tool ‘miRanda’ (<http://www.microrna.org>). psiCHECK2-MRE was constructed by inserting the MRE sequence (139bp) (cDNA obtained by PCR) into the luciferase reporter psiCHECK2 between the Xho1 and Not1 sites. The primer sequences were CGCTCGAGCTCAGAGCTTTGGATCAAGTT (Forward) and TAGCGGCCGCCTG

GCTACAATACTGCACAAA (Reverse). To obtain the psiCHECK2-Mutation vector, point mutagenesis was performed with overlap extension PCR methods on psiCHECK2-MRE vector. The primer sequences were GGCTCATTACCTAAAGCA

GTGGGATTATATGGGATACATCTAATTTTAGAAGCCTGG (Forward) and CCAGGCTTCTAAAATTAGATGTATCCCATATAATCCCACTGCTTTAGGTAATGAGCC (Reverse). PDK1 or H19 cloned from plx304-Blast-V5-PDK1 purchased from GE Healthcare (Buckinghamshire, UK) or WT H19 were inserted in pLVX-DsRed-N1-Monomer (Clontech, Mountain View, CA, USA) between BamH1 and Not1 sites to construct pLVX-PDK1 and pLVX-H19. Lentivirus was packaged in 293T cells using the second-generation packaging system plasmids psPAX2 (Addgene, Cambridge, MA, USA) and pMD2.G (Addgene, Cambridge, MA, USA). One 3.5cm culture dish containing 2×105 HEK293T cells was transfected using Lipofectamine 2000 (Invitrogen, Carlsbad, CA, USA) with 2μg lentiviral vector, 3μg psPAX2 and 1μg pMD2.G Viral particles were collected 48 hours post transfection. After infection, the cells stably expressing H19 were chosen by selection with 2μg/mL puromycin (Sigma, St Louis, MO, USA), respectively. For short hairpin RNA (shRNA) lentiviruses (GenePharma, Suzhou, China) infection, cells were infected in 6-well plates and subsequently split into 10cm dishes in the presence of 2μg/mL puromycin for selection over 72 hours. All sequences were listed in supplementary table 1.

*siRNAs, microRNA mimics and microRNA inhibitors transfection*

Transient transfection was performed by using Lipofectamine 2000 (Invitrogen, Carlsbad, CA, USA) according to the manufacturers’ protocols. The following reagents were used: siRNAs specifically targeting H19, PDK1, HIF1A and siRNA control were purchased from GenePharma and miR-let7a or miR-let7b mimics, Pre-miR negative control, miR-let7a or miR-let7b inhibitors and anti-miR control were purchased from Qiagen, Germany (let-7a, Cat. No. MS00006482, let-7b, Cat. No. MS00003122). All sequences were listed in Supplementary Table 1.

*BrdU staining assay*

Cell proliferation was measured via 5-bromo-2’-deoxyuridine (BrdU) incorporation using a BrdU cell proliferation Detection Kit (KGA319-1; Keygen, Jiangsu, China). Briefly, cells were treated with 30μM BrdU (CB2951; Coolable, Beijing, China) for 30 minutes at 37°C. Subsequently, the cells were washed with phosphate buffered saline (PBS), trypsinized, and quenched with media. After centrifugation, cells were resuspended in washing buffer. Then cells were centrifuged and fixed with 2.5% [paraformaldehyde](javascript:void(0);)at 4°C overnight. Next day, cells were washed with washing buffer, and resuspended in permeation buffer for 2 minutes on ice. Then cells were resuspended in the DNA denaturation solution at 37°C for 30 minutes. Next, cells were resuspended in 195μL dyeing buffer, and incubated with 5μL FITC-BrdU antibody at 4°C for 30 minutes in darkness. Twenty-thousand cells were analyzed using flow cytometry (BD Accuri C6 Plus, Ann Arbor, MI, USA).

*Cell cycle analysis*

Different groups of cells were seeded in 6 cm dishes, then cells were trypsinized with trypsin, quenched with media, washed twice in PBS, and fixed in 75% pre-cold ethanol at 4°C for 4 hours. After RNase A (100μg/mL) (2158; TaKaRa, Dalian, China) digestion at 37°C for 30 minutes, the cells were stained with 50μg/mL PI (P4170; Sigma) for 15 minutes at room temperature before analysis with a flow cytometer (BD Accuri C6 Plus, Ann Arbor, MI, USA).

*Cell viability assay*

Dissociated cells (2×10^3^) were seeded in triplicate in 96-well plates. At day 2, ten microliters of sterile cell counting kit-8 (CCK-8, Beyotime, Shanghai, China) were added to each well and incubated for three hours at 37°C. The optical density values were determined at least in triplicate against a reagent blank at a test wavelength of 450 nm and reference wavelength of 630 nm.

*Immunofluorescent staining*

The frozen sections of xenograft tissue were fixed with 4% formalin for 15 minutes. After rinsing with phosphate buffered saline (PBS, Shanghai Sengon Company, Shanghai, China), sections were permeabilized in 0.25% Triton-X-100 for 15 minutes and blocked with 3% BSA. After rinsing with PBS, non-specific sites on the sections were blocked with 3% BSA for 20 minutes. Sections were then incubated with primary antibodies diluted in 3% BSA for 1 hour: anti-PDK1 (Cell Signaling Technology, Danvers, MA, USA), HIF-1α (Cell Signaling Technology, Danvers, MA, USA), LIN28 (Abcam, #46020, Cambridge, MA, USA), OCT4 (Cell Signaling Technology, Danvers, MA, USA), C-MYC (Cell Signaling Technology, Danvers, MA, USA). After rinsing with PBS, the sections were incubated with Alexa Fluor 488 Goat Anti-Rabbit IgG (H+L) (Invitrogen, Carlsbad, CA, USA) for 1 hour in the dark. The sections were rinsed by PBS, stained with 100ng/ml 4′, 6-diami-dino-2-phenylindole (DAPI, Thermo Fisher Scientific, Rockford, lL, USA) for 5 minutes, and rinsed with PBS. The sections were dried and placed on embedding medium on cover slides, and then observed under confocal microscope (Leica, Wetzlar, Germany). Images from each experiment were acquired using the same exposure time during the same imaging session, total populations and positive cells were measured by Image-Pro Plus software.

*Hematoxylin and Eosin staining of tissue sections*

Paraffinembedded tumor xenografts tissue specimens were sectioned, deparaffinized in xylene and rehydrated. Dip the slide into a coplin jar containing hematoxylin (Beyotime, Shanghai, China) and agitate for 30 seconds. After rinse the slide in H_2_O, stain the slide with 1% eosin Y (Beyotime, Shanghai, China) solution for 30 seconds with agitation. Dehydrate the sections with two changes of 95% alcohol and two changes of 100% alcohol for 30 seconds each. Extract the alcohol with two changes of xylene. Add one or two drops of mounting medium and cover with a coverslip.

*Luciferase reporter assay*

MDA-MB-231 cells (1×10^4^) were seeded into each well of a 48-well-plate and the following steps were carried out as previously described.^4^ MDA-MB-231 cells were co-transfected with 10 ng of the indicated luciferase reporter and 48nM miRNA mimics (Qiagen, Valencia, CA, USA) using Lipofectamine 2000 (Invitrogen, Carlsbad, CA, USA). Eighteen hours after transfection, luciferase activity was monitored using the Dual-Luciferase Reporter Assay System (Promega, Madison, WI, USA) and a luminometer (Molecular Devices, Sunnyvale, CA, USA). Renilla luciferase activity was normalized against firefly luciferase activities and presented as percentage of inhibition. Results represented the average of triplicate samples from three independent experiments.

**References**

1. Zheng F, Yue C, Li G, He B, Cheng W, Wang X *et al*. Nuclear AURKA acquires kinase-independent transactivating function to enhance breast cancer stem cell phenotype. *Nat Commun* 2016; **7:** 10180.
2. Kallen AN, Zhou XB, Xu J, Qiao C, Ma J, Yan L *et al*. The imprinted H19 lncRNA antagonizes let-7 microRNAs. *Mol Cell* 2013; **52:** 101-12.
3. Iwasaki S, Kawamata T, Tomari Y. Drosophila argonaute1 and argonaute2 employ distinct mechanisms for translational repression. *Mol Cell* 2009; 34: 58-67.
4. Qiu C, Ma Y, Wang J, Peng S, Huang Y. Lin28-mediated post-transcriptional regulation of Oct4 expression in human embryonic stem cells. *Nucleic Acids Res* 2010; **38:** 1240–8.
