# Supplementary figures and images for: Glycolysis gatekeeper PDK1 reprograms breast cancer stem cells under hypoxia
Source: Oncogene. 2017 Nov 6;37(8):1062–74. doi: 10.1038/onc.2017.368 (PMC5851116; doi:10.1038/onc.2017.368)

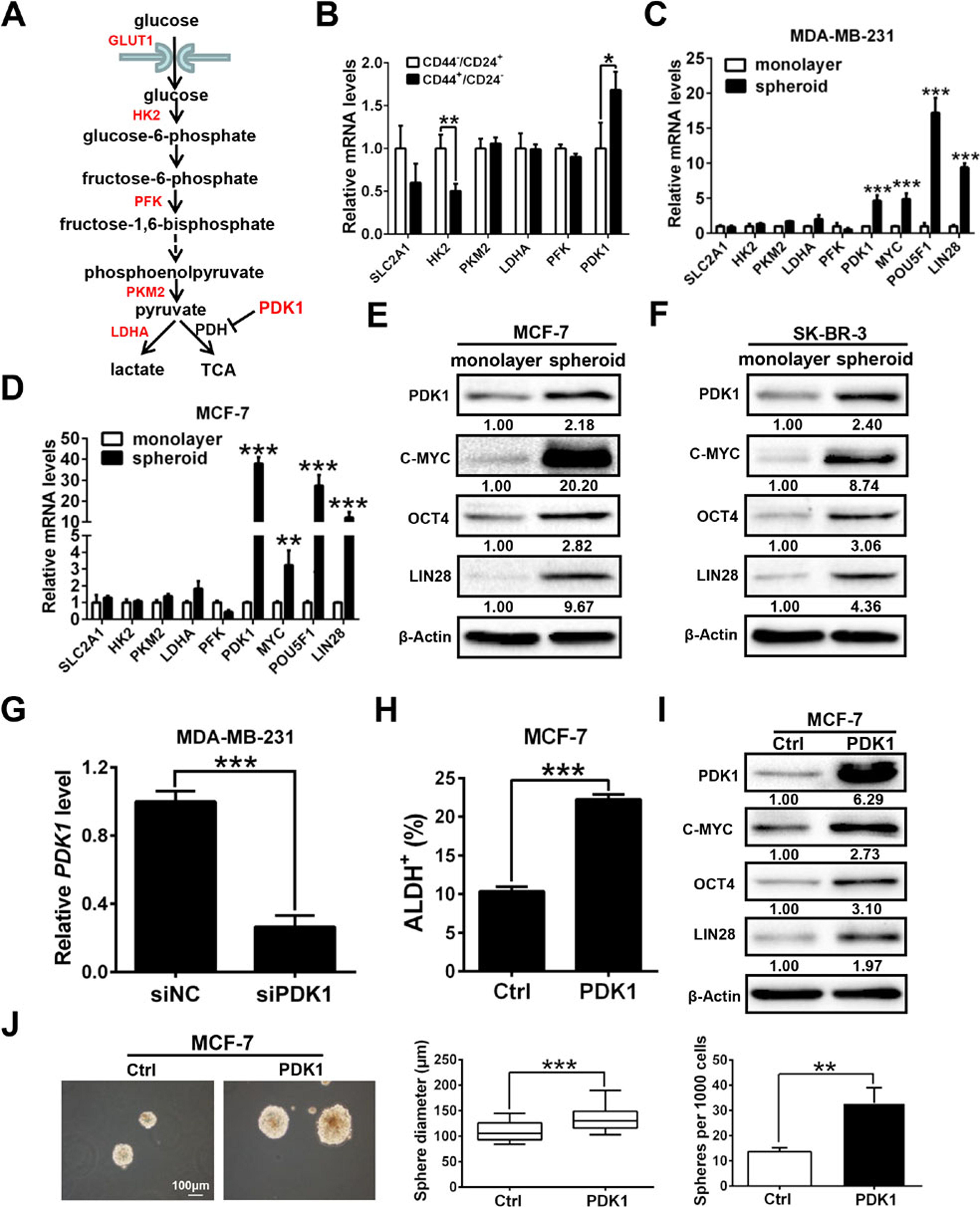

Supplement: Supplementary Figure 1 [file onc2017368x2.tif]

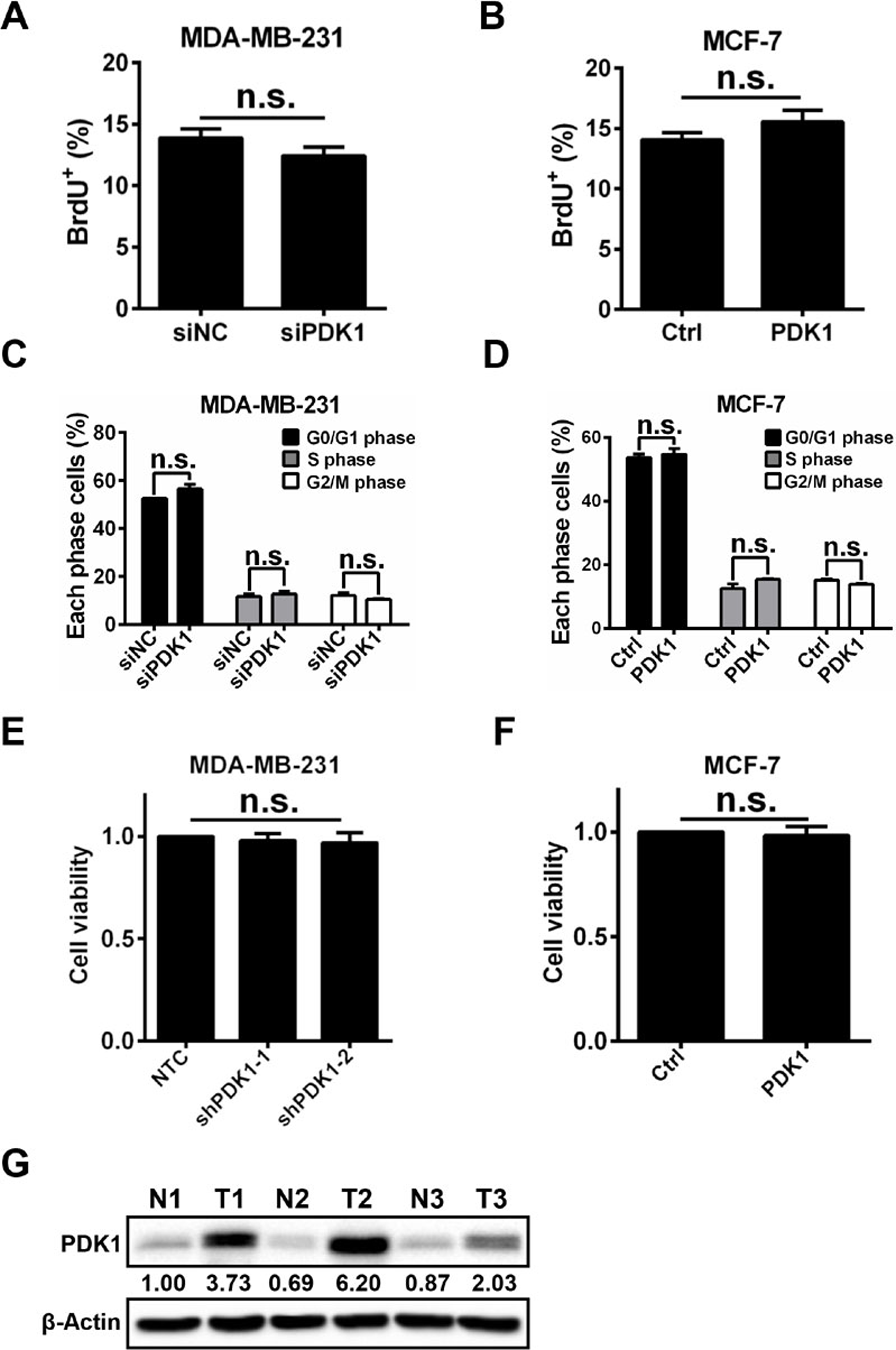

Supplement: Supplementary Figure 2 [file onc2017368x3.tif]

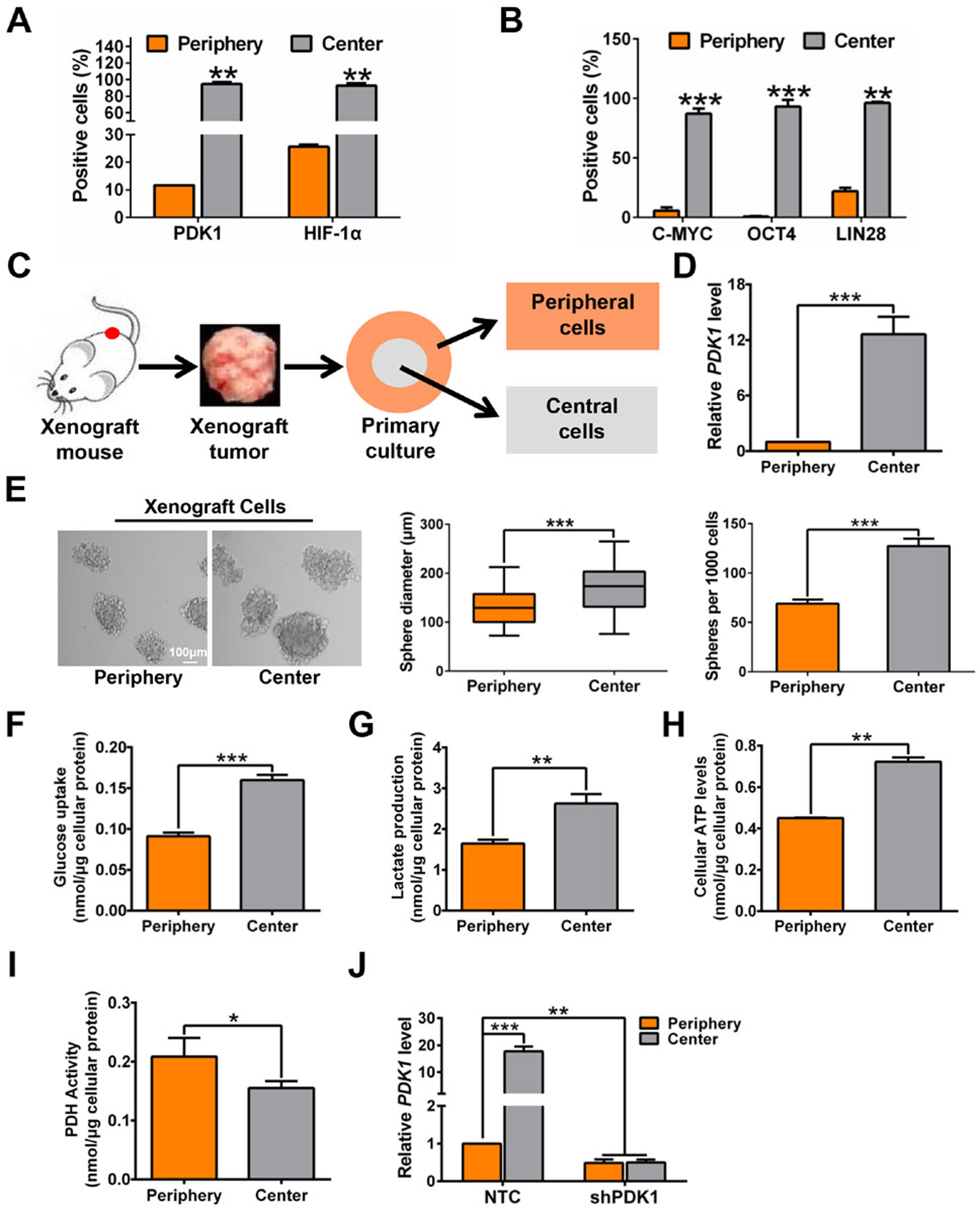

Supplement: Supplementary Figure 3 [file onc2017368x4.tif]

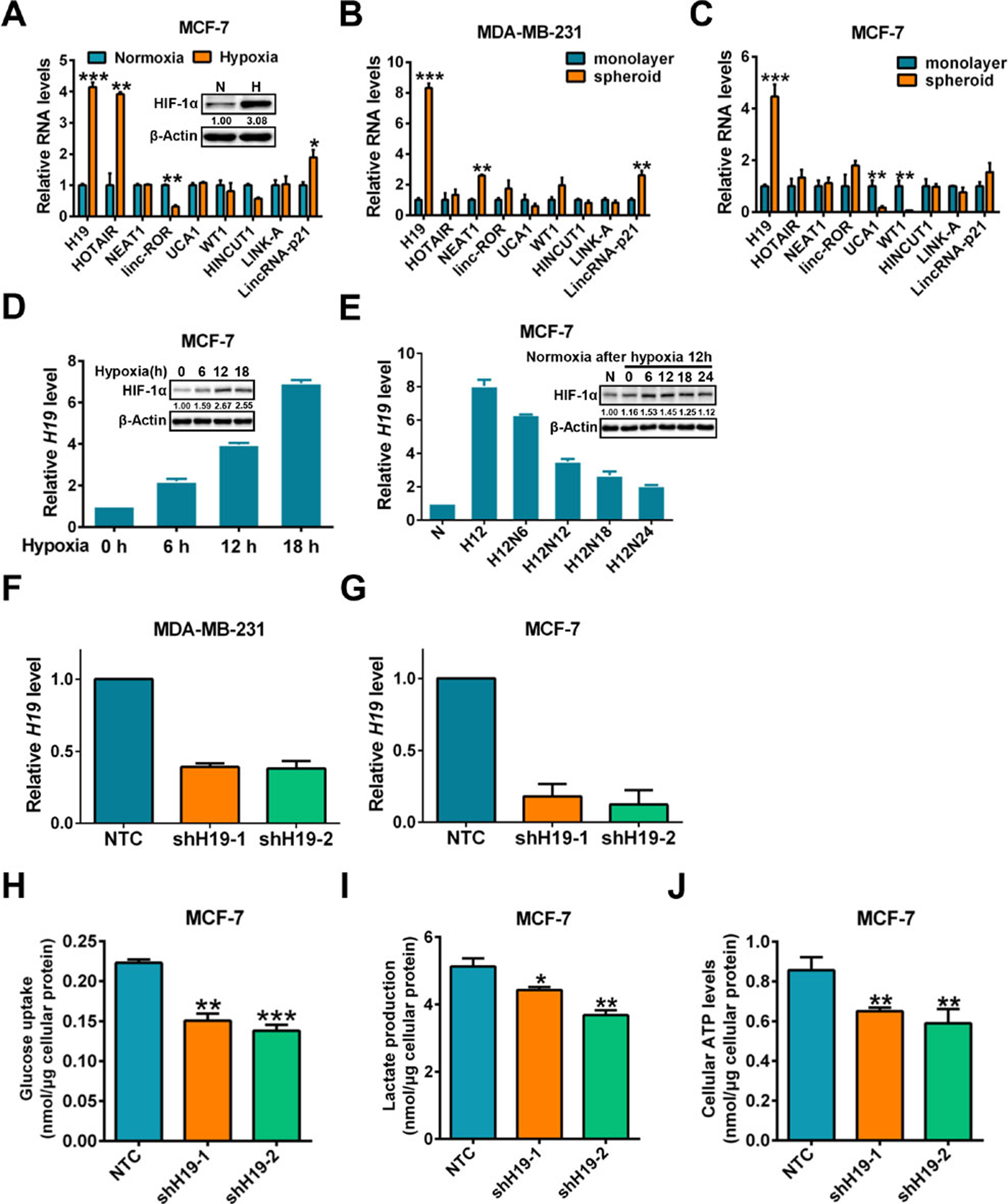

Supplement: Supplementary Figure 4 [file onc2017368x5.tif]

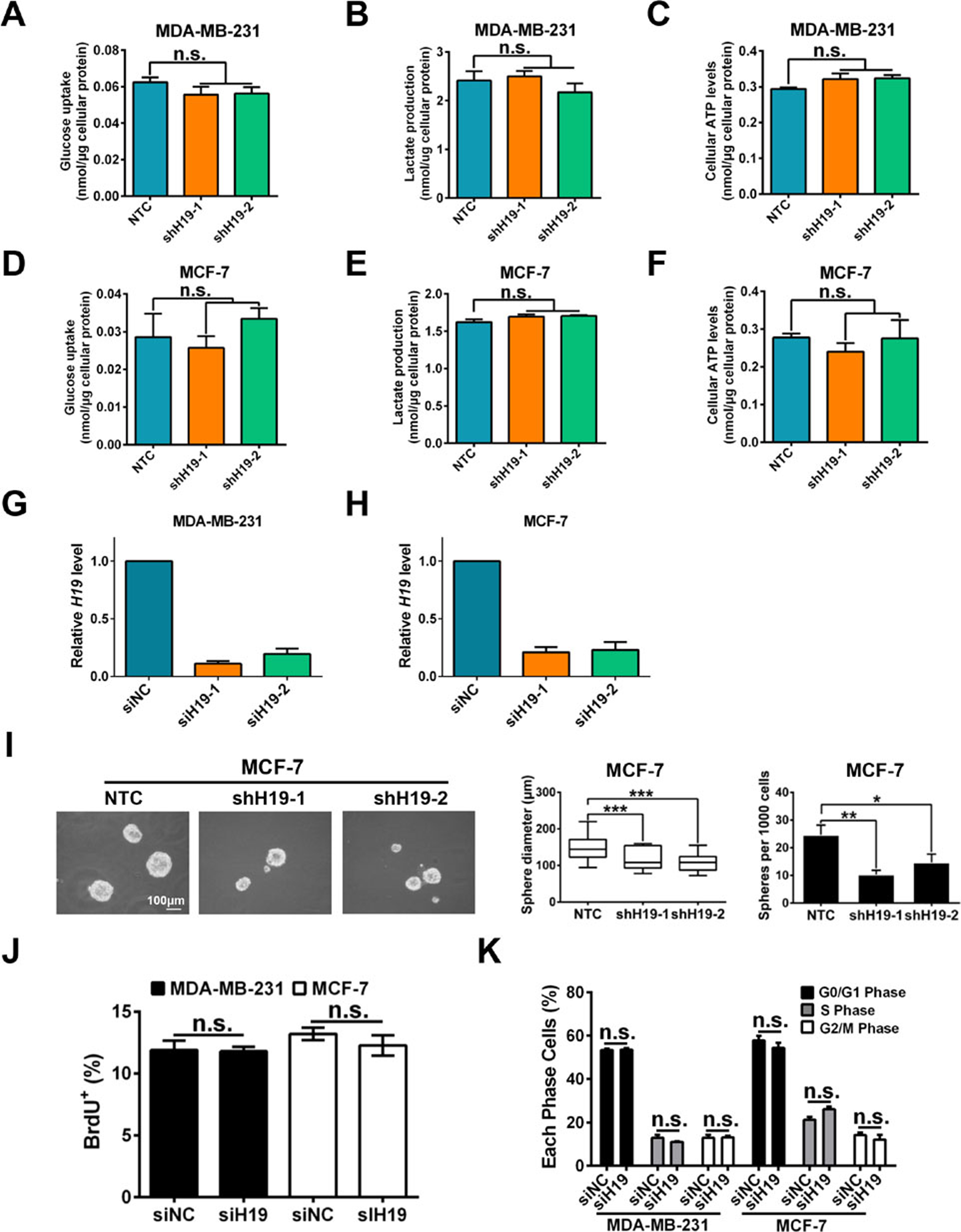

Supplement: Supplementary Figure 5 [file onc2017368x6.tif]

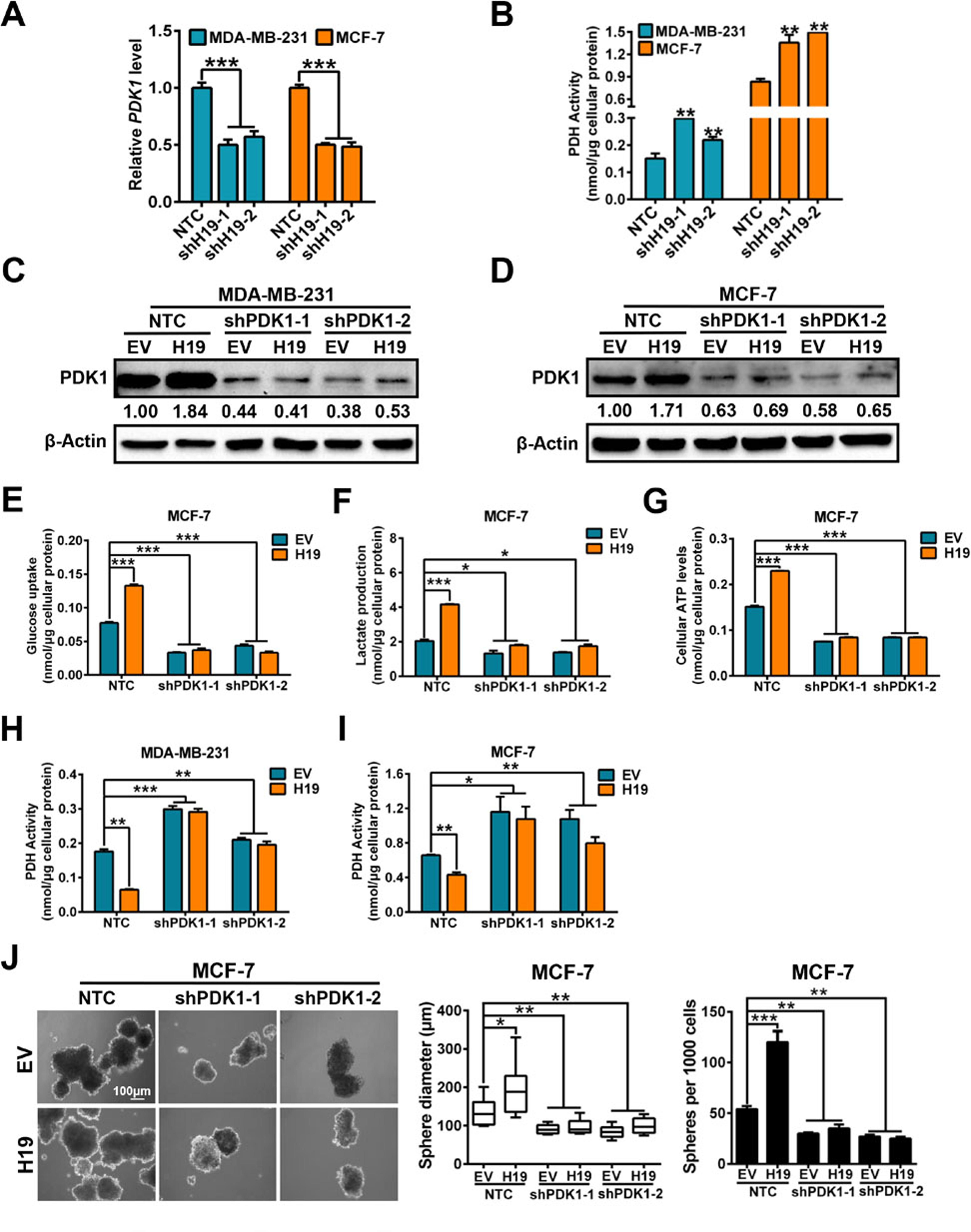

Supplement: Supplementary Figure 6 [file onc2017368x7.tif]

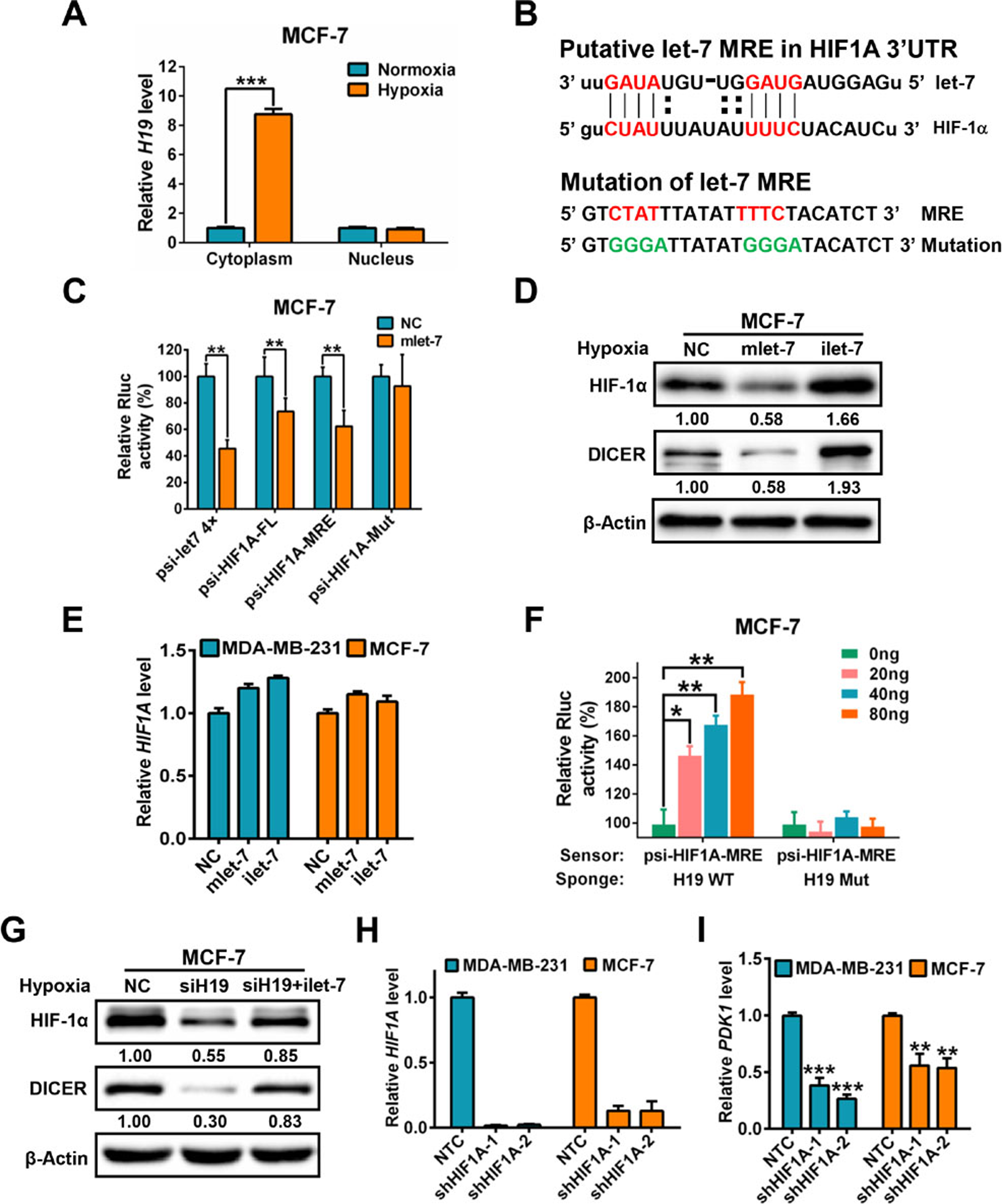

Supplement: Supplementary Figure 7 [file onc2017368x8.tif]

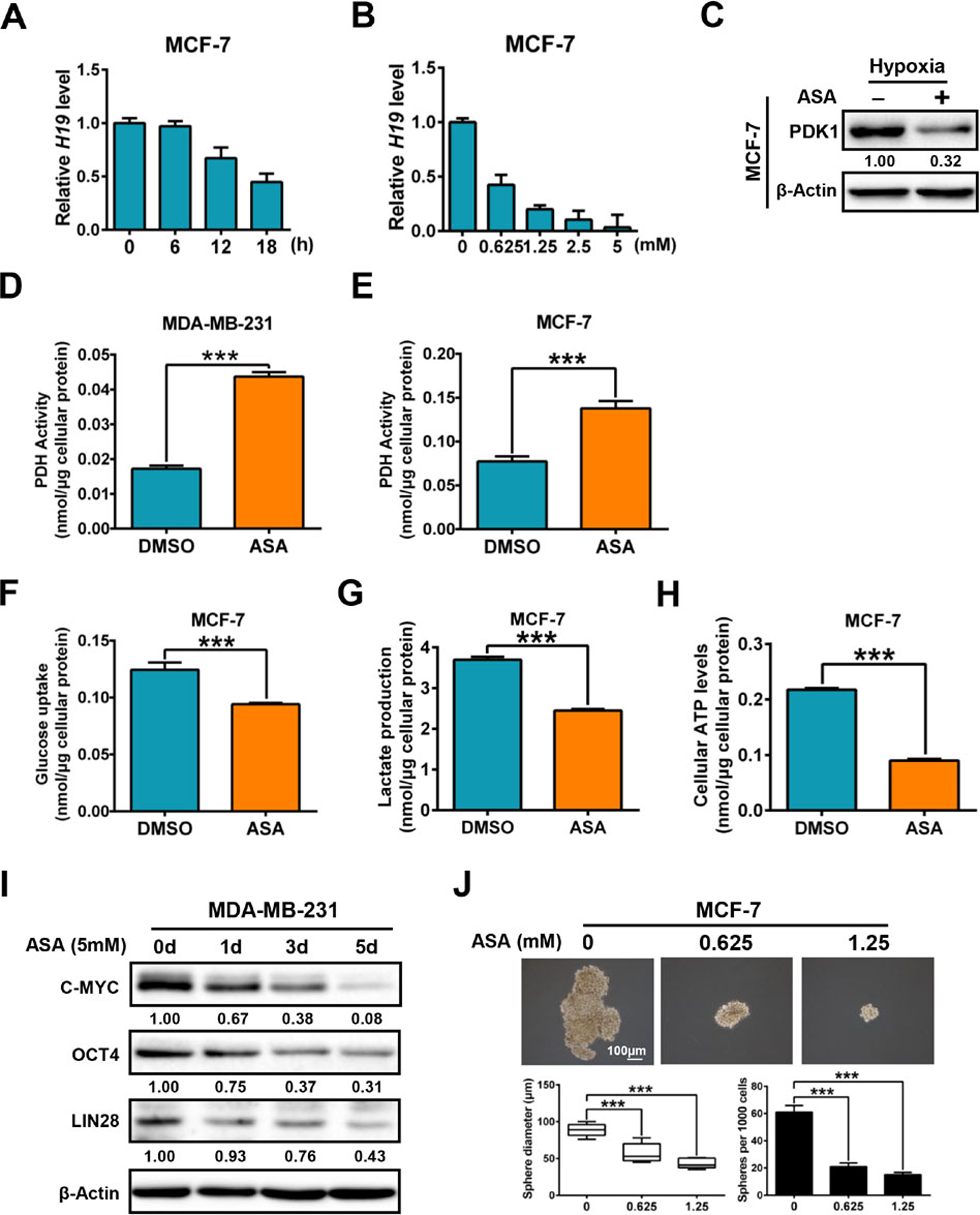

Supplement: Supplementary Figure 8 [file onc2017368x9.tif]
