# Supplementary material for: Glycolysis gatekeeper PDK1 reprograms breast cancer stem cells under hypoxia
Source: Oncogene. 2017 Nov 6;37(8):1062–74. doi: 10.1038/onc.2017.368 (PMC5851116; doi:10.1038/onc.2017.368)
Supplement: Supplementary Table 1 [file onc2017368x10.pdf]

The primers used in quantitative PCR are shown

Genes	Forward Primer 5′ ＞3′	Reverse Primer 5′ ＞3′
ACTB	ATCAAGATCATTGCTCCTCCTGAG	CTGCTTGCTGATCCACATCTG
PDK1	ACTTCGGATCAGTGAATGCTTG	ACTCTTGCCGCAGAAACATAAA
ALDH1	ATCAAAGAAGCTGCCGGGAA	GCATTGTCCAAGTCGGCATC
H19	GCACCTTGGACATCTGGAGT	TTCTTTCCAGCCCTAGCTCA
POU5F1	GTGGAGGAAGCTGACAACAA	GCCGGTTACAGAACCACACT
SOX2	GGTTACCTCTTCCTCCCACTCC	CCCTCCCATTTCCCTCGTTT
NANOG	ACCTATGCCTGTGATTTGTGG	AGTGGGTTGTTTGCCTTTGG
MYC	CGAGGAGGAGAACTTCTACCAGC	CGAGAAGCCGCTCCACATACAGTCC
HOTAIR	CAGTGGGGAACTCTGACTCG	GTGCCTGGTGCTCTCTTACC
lncRoR	CTGGCTTTCTGGTTTGACG	CAGGAGGTTACTGGACTTGGAG
NEAT1	CCAGTTTTCCGAGAACCAAA	ATGCTGATCTGCTGCGTATG
UCA1	CTCTCCATTGGGTTCACCATTC	GCGGCAGGTCTTAAGAGATGAG
WT1	GCCTCTCTGTCCTCTTCTTTGT	GCTGTGAGTCCTGGTGCTTAGC
HINCUT1	CAGGTGGGTGAAACCTCCTA	TGGAAGAGGAGGAGGAGGTT
LINK-A	TTCCCCCATTTTTCCTTTTC	CTCTGGTTGGGTGACTGGTT
LincRNA-p21	CCTGTCCCACTCGCTTTCCATT	GGAACTGGAGACGGAATGTC
HIF1A	GAACGTCGAAAAGAAAAGTCTCG	CCTTATCAAGATGCGAACTCACA

shRNA sequences

Name	5′ >3′
**shPDK1-1**	GGAAGGATACGGACCTCTTAA
**shPDK1-2**	GCCTAACAGGACGTATTATCT
**shHIF1α-1**	GCCGAGGAAGAACTATGAACA
**shHIF1α-2**	GGTATAAGAAACCACCTATGA
**shH19-1**	GCACTACCTGACTCAGGAATC
**shH19-2**	GACCTCATCAGCCCAACATCA
**NTC**	TTCTCCGAACGTGTCACGTTTC

siRNA sequences

Name	Sense 5′ >3′	Anti-sense 5′ >3′
**siH19-1**	CCCGUCCCUUCUGAAUUUATT	UAAAUUCAGAAGGGACGGGTT
**siH19-2**	GCGGGUCUGUUUCUUUACUTT	AGUAAAGAAACAGACCCGCTT
**siPDK1**	GGAUGCUAAAGCUAUUUAUTT	AUAAAUAGCUUUAGCAUCCTT
**siNC**	UUCUCCGAACGUGUCACGUTT	ACGUGACACGUUCGGAGAATT

microRNA mimics sequences

Name	Sense 5′ >3′	Anti-sense 5′ >3′
let-7a	UGAGGUAGUAGGUUGUAUAGUU	CUAUACAACCUACUACCUCAUU
let-7b	UGAGGUAGUAGGUUGUGUGGUU	CCACACAACCUACUACCUCAUU

microRNA inhibitors sequences

Name	5′ >3′
let-7a	AACUAUACAACCUACUACCUCA
let-7b	AACCACACAACCUACUACCUCA

Information of 15 breast cancer tissues

Case	Age	Sex	TNM	Stage	ER	PR	HER2
110046	46	F	T2N1M1	II A	-	-	-
112789	50	F	T4N2M0	I B	+	+	-
112378	37	F	T3N3M0	II B	-	-	-
123456	52	F	T2N0M0	I A	++	++	++
117891	61	F	T2N1M0	II B	+++	+	+
111457	56	F	T1N0M0	II A	-	-	+
127560	70	F	T3N2M0	II B	+	+	-
113589	42	F	T1N1M0	I A	+++	++	+
120014	51	F	T4N1M1	III A	+	+	-
114567	65	F	T2N1M0	II A	+++	++	-
124789	56	F	T2N2M0	II B	-	-	+
120421	48	F	T2N1M0	II B	-	-	+/-
112035	60	F	T3N1M0	II A	-	-	-
121116	58	F	T2N2M1	II B	-	-	+/-
117891	56	F	T2N3M1	III A	-	-	-
